# Identification and development of a subtype-selective allosteric AKT inhibitor suitable for clinical development

**DOI:** 10.1038/s41598-022-20208-5

**Published:** 2022-09-20

**Authors:** Natalie Page, Mark Wappett, Colin R. O’Dowd, Martin O’Rourke, Gerald Gavory, Lixin Zhang, J. S. Shane Rountree, Linda Jordan, Oliver Barker, Hayley Gibson, Caroline Boyd, Stephanie Feutren-Burton, Estelle McLean, Graham Trevitt, Timothy Harrison

**Affiliations:** 1 Almac Discovery Ltd, Health Sciences Building, 97 Lisburn Road, Belfast, BT9 7AE Northern Ireland UK; 2 Patrick G Johnston Centre for Cancer Research, Queen’s University Belfast, Belfast, BT9 7AE Northern Ireland UK; 3 Present Address: Sygnature Discovery, BioCity, Pennyfoot Street, Nottingham, NG1 1GR UK; 4 Present Address: Amphista Therapeutics, BioCity, Bo’Ness Rd, Newhouse, Chapelhall, Motherwell, ML1 5UH UK; 5 Present Address: Ridgeline Therapeutics GmbH, Technologiepark, Hochbergerstrasse 60C, 4057 Basel, Switzerland; 6 Present Address: Globachem, Alderley Park, 2 BioHub, Mereside, Macclesfield, SK10 4TG UK; 7 Present Address: Shenyang University of Chemical Technology, Shenyang, China

**Keywords:** Drug discovery, Oncology

## Abstract

The serine/threonine protein kinase AKT plays a pivotal role within the PI3K pathway in regulating cellular proliferation and apoptotic cellular functions, and AKT hyper-activation via gene amplification and/or mutation has been implicated in multiple human malignancies. There are 3 AKT isoenzymes (AKT1-3) which mediate critical, non-redundant functions. We present the discovery and development of ALM301, a novel, allosteric, sub-type selective inhibitor of AKT1/2. ALM301 binds in an allosteric pocket created by the combined movement of the PH domain and the catalytic domain, resulting in a DFG out conformation. ALM301 was shown to be highly selective against a panel of over 450 kinases and potently inhibited cellular proliferation. These effects were particularly pronounced in MCF-7 cells containing a PI3KCA mutation. Subsequent cellular downstream pathway analysis in this sensitive cell line revealed potent inhibition of pAKT signalling up to 48 h post dosing. ALM301 treatment was well tolerated in an MCF-7 xenograft model and led to a dose-dependent reduction in tumour growth. Enhanced efficacy was observed in combination with tamoxifen. In summary, ALM301 is a highly specific AKT 1/2 inhibitor with an excellent pharmacological profile suitable for further clinical development.

## Introduction

AKT is a serine/threonine kinase which plays a key role in the phosphatidylinositol-3-kinase (PI3K)/AKT/mammalian target of rapamycin (mTOR) signalling pathway and is involved in cell signalling, proliferation, glucose metabolism and protein synthesis via phosphorylation of two critical residues, threonine 308 and serine 473^1,2^. Hyper-phosphorylation of AKT in response to specific inhibitor treatment can result in a non-functional state by preventing access of phosphatases^3^. There are 3 AKT isoenzymes (AKT1-3) which mediate critical non-redundant functions; thus the possibility exists to target specific subtype combinations in particular clinical indications^4^.

The PI3K/AKT/mTOR pathway is one of the most frequently mutated pathways in cancer and aberrant activation of the pathway has been linked to several human malignancies and poor prognosis in many tumour types including pancreatic, ovarian, breast and head and neck cancers^5–10^. Intra-tumoral activation of AKT has been correlated with resistance to multiple classes of inhibitors including EGFR inhibitors, estrogen receptor antagonists and aromatase antagonists in breast cancer and anti-hormone therapies in prostate cancer^11–13^. Combination of AKT inhibition with the therapies outlined above has been shown to re-sensitize tumours to conventional therapies^14^.

Small molecule AKT inhibitors are typically either ATP competitive or allosteric with respect to their binding site. Allosteric inhibitors induce a conformational change through binding to the pleckstrin homology (PH) domain, thus preventing localisation of AKT to the plasma membrane and therefore inhibiting activation of the pathway. Consequently, these inhibitors tend to show higher selectivity for AKT over other kinases, as well as circumventing feedback mechanisms resulting from hyper-phosphorylation of AKT, which are commonly observed following treatment with ATP competitive inhibitors. Several AKT inhibitors have been advanced to clinical trials, including GSK 2110183 (afuresertib, Phase 3, breast cancer), AZD5363 (capivasertib, Phase 3, prostate and breast cancer) and GDC-0068 (ipatasertib, Phase 3, prostate and breast cancer). These inhibitors are all ATP competitive inhibitors which target the active form of the kinase. Additionally, a number of allosteric AKT inhibitors have also entered clinical trials (e.g. MK-2206 (Phase 2, multiple cancer indications), ARQ-092 (miransertib, Phase 2, CLOVES syndrome), and TAS-117 (Phase 2, PTEN mutant cancers)) with MK-2206 being the most clinically advanced allosteric AKT inhibitor^15–19^. Other small molecule allosteric AKT inhibitors such as borussertib (which binds covalently to non-catalytic cysteines in AKT) have shown promise in preclinical studies but as yet have not progressed into clinical studies^20,21^. Since a range of clinical toxicities (including hyperglycaemia and skin rash) have been reported during the clinical development of a number of these compounds, new compounds (with different sub-type selectivities, off-target and ADME profiles) may offer the opportunity to improve the therapeutic index^22–24^.

Herein we report the development and characterization of ALM301, a novel subtype-selective allosteric small molecule inhibitor of AKT.

## Results

### Identification of ALM301

**Figure 1:**
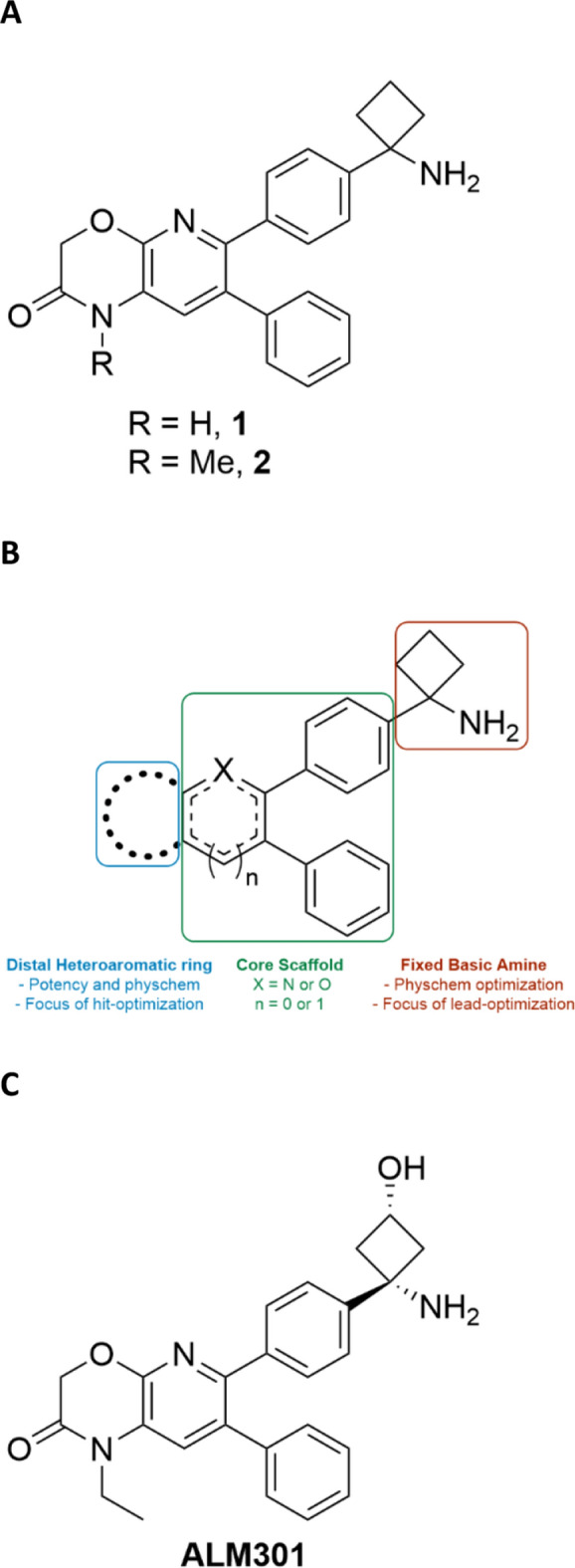
Early development of novel allosteric AKT inhibitors based on a bicyclic pyrido-oxazinone core. (**A**) Chemical structures of early lead chemotypes used as starting points for medicinal chemistry development. (**B**) Hit and lead-optimization strategies employed. (**C**) Chemical structure of development candidate ALM301.

Compound 1 (Fig. 1A) was identified as a promising and ligand efficient early lead following a focussed medicinal chemistry campaign aimed at identifying novel allosteric AKT inhibitors. Our early medicinal chemistry strategy centred on scaffold-hopping and optimization of key structural elements of known prototypical allosteric AKT inhibitors. As such, a strand of our medicinal chemistry program involved optimisation of the fused bicyclic ring motif as shown in Fig. 1B. Multiple fused bicyclic cores were investigated during the course of the hit-to-lead chemistry phase with the pyrido-oxazinone motif (as exemplified by compound 1) being the most promising in terms of overall potency and physicochemical properties. Compound 1 demonstrated moderate biochemical activity across AKT isoforms with IC_50_ values ranging from 2.5 to 6.1 µM and served as a starting point for further medicinal chemistry optimization strategies, which were primarily aimed at improving AKT biochemical potency and overall physicochemical properties (Table 1). These strategies included focussed analogue design and SAR analyses centred on the distal oxazinone ring of compound 1, whilst maintaining the core 2,3-diphenyl pyrido-scaffold. Methylation of the oxazinone nitrogen atom in compound 1 afforded compound 2 which demonstrated a significant improvement in biochemical potency versus AKT1/2 (Fig. 1A and Table 1). The improved biochemical potency of compound 2 is most likely due to general hydrophobic and van der Waals effects of the additional *N*-methyl group, based on inspection of docked binding poses of both compounds 1 and 2 in published AKT crystal structures. In addition to excellent kinetic solubility (> 190 µM), compound 2 produced a marked decrease in phosphorylation of the AKT substrate pGSK3β in PC3 prostate cancer cells, with a potency improvement of ca. 30-fold when compared with the des-*N*-methyl analogue 1.

**Table 1:** Potency and physicochemical characterization of initial leads.

	1	2
**AKT IC**_**50**_** (µM)**		
AKT1	2.5	0.1
AKT2	3.3	0.4
AKT3	6.1	5.6
PC3, pGSK3β ELISA IC_50_ (µM)	3.0	0.1
Kinetic solubility pH_7.4_ (µM)	206	191
MW/clogP	371/3.8	385/3.3
AKT1 ligand efficiency (LE)	0.28	0.34

The next phase of our medicinal chemistry program involved further iterative changes to the distal oxazinone ring, with focus shifting to analogues of the basic amine moiety in the latter stages of the program (Fig. 1B). Further chemistry optimization focussing on the chemotype exemplified by compound 2 (Fig. 1A) afforded clinical development candidate ALM301 (Fig. 1C and Table 2). ALM301 has a superior overall profile when contrasted with earlier lead molecules demonstrating potent inhibition of AKT1/2 in biochemical assays, excellent kinome selectivity, potent anti-proliferative effects in sensitive cell lines and a good pharmacokinetic profile in rat (Table 2). Additionally, ALM301 also demonstrated improved pAKT inhibition in MCF7 cells, increased oral bioavailability in rat and a reduced potential liability versus the 3A4 cytochrome P450 (CYP3A4) (with respect to time-dependent inhibition of this important metabolizing enzyme) when benchmarked against the most advanced clinical allosteric AKT inhibitor, MK-2206 (Table 2 and Supplementary Fig. S3C).

**Table 2:** Potency, physicochemical and early ADME profiling of ALM301 and MK-2206.

	ALM301	MK-2206
**AKT IC**_**50**_** (µM)**		
AKT1	0.13	0.02
AKT2	0.09	0.06
AKT3	2.75	0.18
PC3, pGSK3β ELISA IC_50_ (µM)	0.16	0.12
MW / PSA (Å^2^)	415.5/88	407.5/84
Kinetic solubility pH_7.4_ (µM)	192	190
logD_7.4_	1.2	2.4
h/r/m LM CL_int_ (µL/min/mg protein)	3.7/3.0/6.0	20.0/13.0/N.D
CYP IC_50_ (3A4, 2D6, 1A2, 2C9, 2C19) (µM)	All > 25	3A4^#^ 12; 2C9 19
Rat PK*		
CL (mL/min/kg)	19	24
*V*_d_ (L/kg)	13	12
*F* (%)	26	12

LM: Liver microsomes.

*Male SD rats at 1 mg/kg *i.v.* and 5 mg/kg p.o. PK parameters calculated based on AUC 0-∞. ^#^ > 2.7 fold Time Dependent Inhibition (TDI) of CYP3A4 observed.

### ALM301 is a potent allosteric inhibitor of AKT1 and AKT2

**Figure 2:**
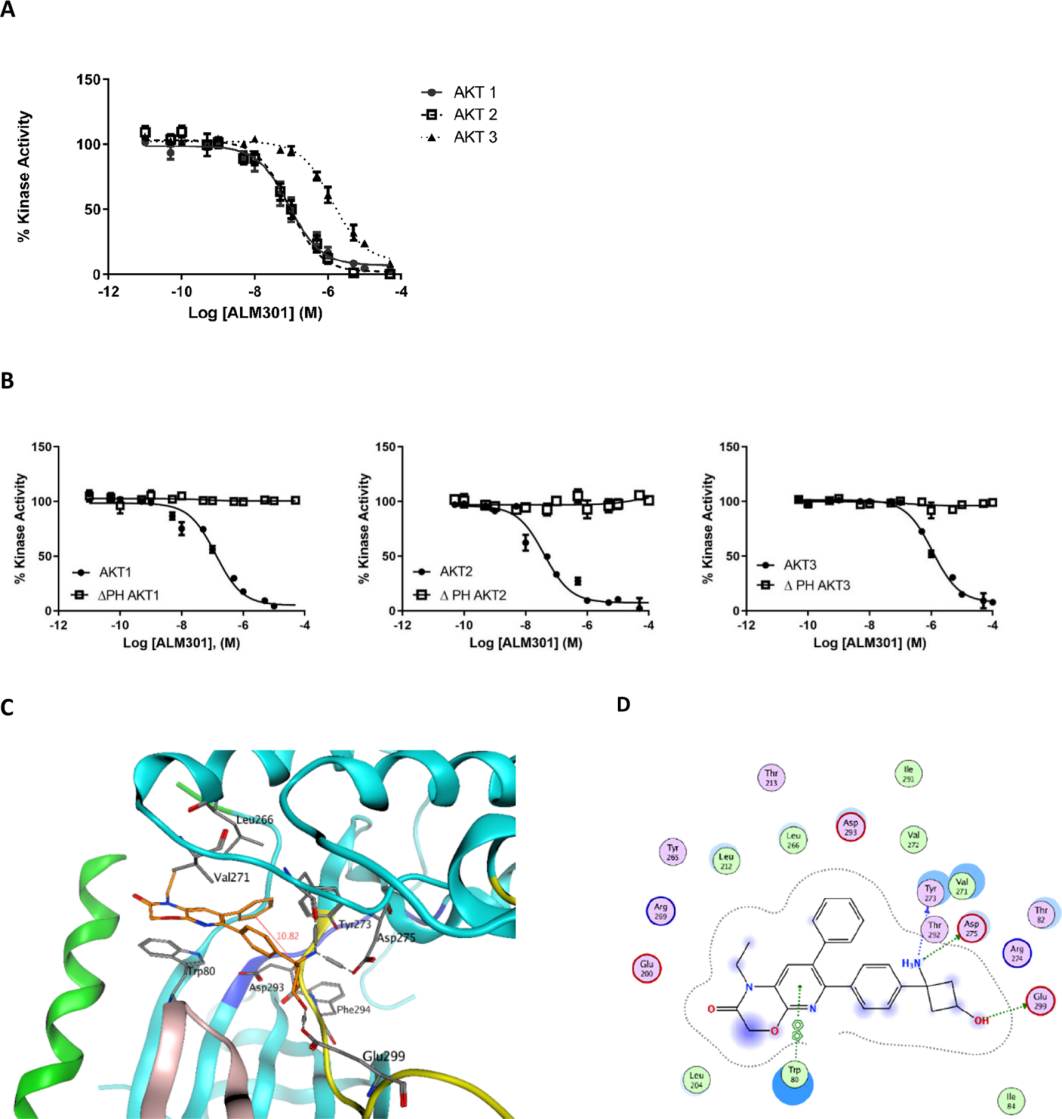
Biochemical inhibition of AKT isoforms versus pH domain null isoforms and ALM301 docking studies. (**A**) Biochemical inhibition of AKT1/AKT2/AKT3 and (**B**) related PH domain null isoforms using ALM301. (**C**) Docked pose of ALM301 in the allosteric pocket of AKT2 based on the crystal structure data generated using the closely related analogue compound 3. (**D**) Interaction diagram based on the docking of ALM301 with the allosteric pocket of AKT2 depicting key amino acid interactions.

ALM301 potently and selectively inhibited the activity of full length AKT1 and AKT2 in a biochemical kinase assay, with IC_50_ values of 125 nM and 95 nM respectively (compared to > 500 nM against AKT3) (Fig. 2A). The truncated forms of all three AKT isoforms lacking PH domains were not inhibited (Fig. 2B). Incubating ALM301 with increasing concentrations of ATP had no effect on the biochemical IC_50_ value versus AKT1, confirming that the mode of binding for ALM301 is allosteric and independent of ATP concentration (Supplementary Table S1). The allosteric binding mode of this series was confirmed via a high resolution (2.32 Å) co-crystal structure obtained with a close analogue of ALM301 (compound 3) in complex with AKT2 (Supplementary Fig. S1A–C and Tables S3, S4). As shown in Supplementary Fig. S1B, C and D, compound 3 binds in an allosteric binding pocket ca. 10 Å away from the hinge backbone (dark blue) at the interface of the N- and C-lobes of the kinase and PH domains, similar to previously published allosteric AKT inhibitors^25^. The crystal structure showed compound 3 binding to a DFG-out conformation of AKT2, restricting access to the ATP binding site. ALM301 adopted a similar binding mode to compound 3 when docked into the compound 3 binding site (Fig. 2C,D). The bicyclic moiety of ALM301 was predicted to stack against Trp80 with the carbonyl group close to the Arg209 sidechain but not forming a direct hydrogen bond. The unsubstituted phenyl ring occupies the hydrophobic pocked under the DFG loop that would be occupied by Phe294 when the protein adopts a DFG-in conformation. The docked pose places the para-substituted phenyl pointing towards the catalytic subunit, with the polar substituents both forming interactions with the protein; the basic nitrogen forms a salt bridge with Asp275, and the hydroxyl a hydrogen bond with the Glu299 sidechain.

### ALM301 demonstrates excellent selectivity against 450 kinases

ALM301 was shown to be highly selective (at a fixed concentration of 10 µM) against a panel of over 450 kinases (Supplementary Fig. S2A)—notably only two other kinases demonstrated more than 70% inhibition; p38α and PDK1 showing 84% and 77% inhibition respectively (Supplementary Table S2). Further analysis via 10-point titration curve confirmed that the IC_50_ for both p38α and PDK1 was found to be greater than 10 µM (Supplementary Fig. S2B).

### ALM301 inhibits proliferation in a cancer cell line panel

**Figure 3:**
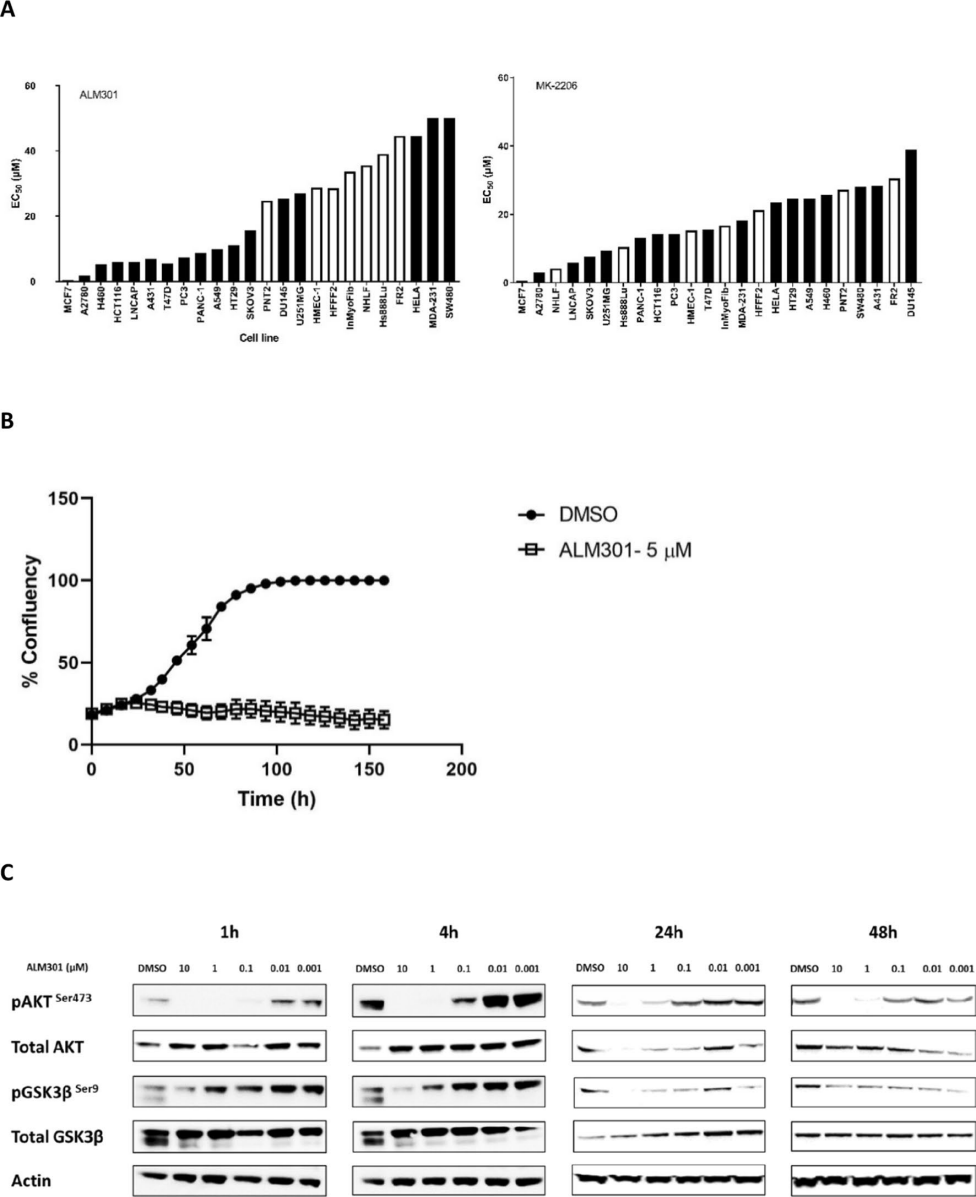

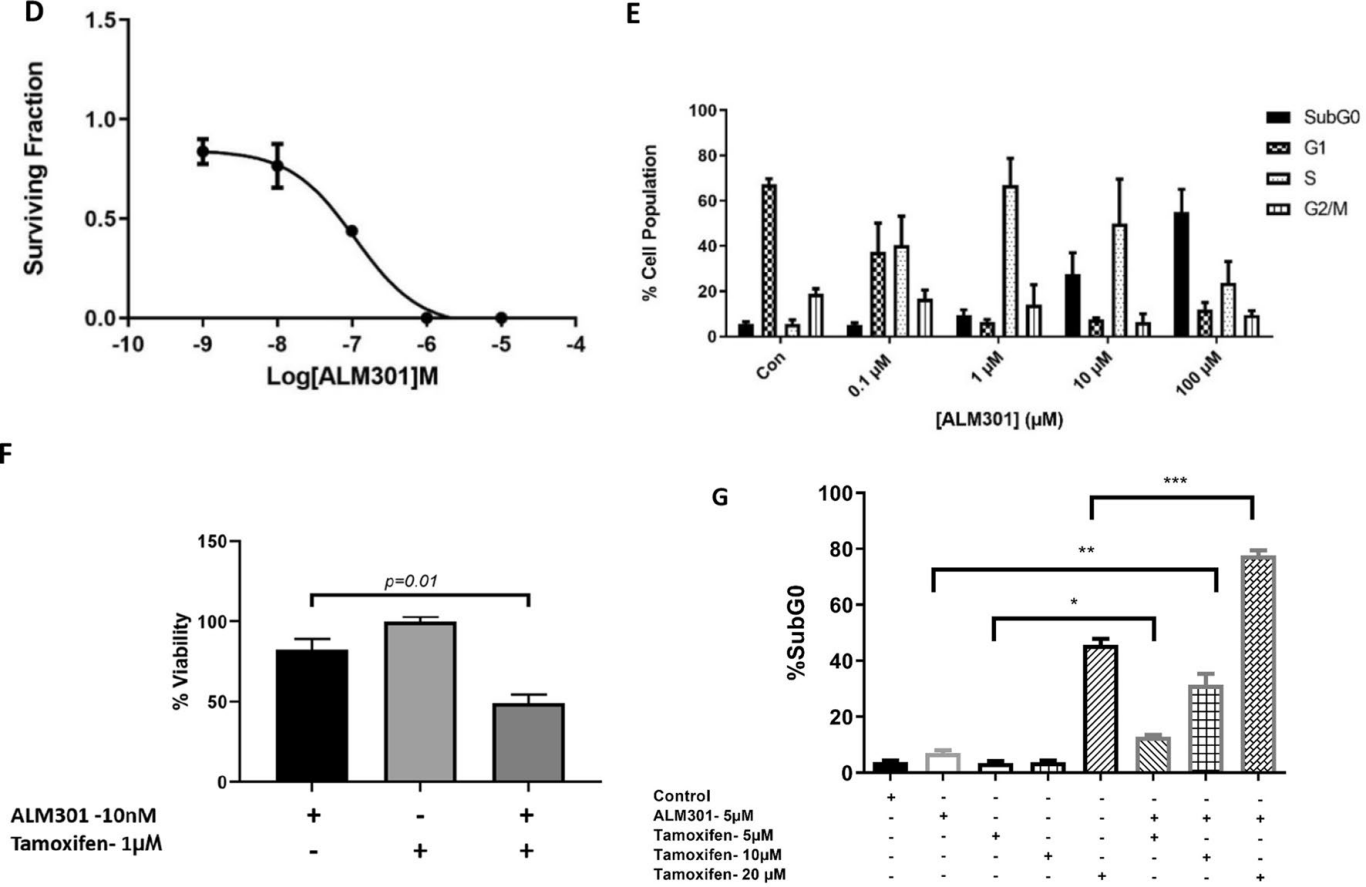
Cellular profiling and validation of development candidate ALM301 in MCF-7 cells both as a single agent and in combination with tamoxifen. (**A**) Cell line profiling of ALM301 and MK-2206 in a panel of 24 cell lines including normal and tumour cells. (Black = cancer cell lines, White = normal cell lines). (**B**) Inhibition of MCF-7 cellular proliferation with 5 µM of ALM301 over 7 days using live cell imaging with an Incucyte imaging system. (**C**) Western blotting demonstrating inhibition of pAKT and pGSK3β in MCF-7 cells at various concentrations and timepoints up to 48 h. (**D**) Clonogenic cell survival of MCF-7 cells treated with ALM301 over a concentration range of 0.001–10 µM (IC_50_ = 100 nM in this assay). (**E**) Cell cycle flow cytometry of MCF-7 cells treated with concentration range of 0.1–100 µM of ALM301 for 72 h. (**F**) Inhibition of MCF-7 cell viability using a combination of ALM301 and Tamoxifen at concentrations of 10 nM and 1 µM respectively. (**G**) Cell cycle flow cytometry of MCF-7 cells treated with ALM301 in combination with tamoxifen indicating apoptotic subG0 cell populations. Paired *t*-test was used to derive *p* values (n = 10/group), *p < 0.05, **p < 0.01, ***p < 0.001.

The anti-proliferative effects of ALM301 were determined in a panel containing both normal and cancer cell lines (including breast, lung and prostate (Fig. 3A)). Normal cells (indicated in white) were amongst the most resistant cell lines in response to ALM301 treatment (IC_50_ > 20 µM). MCF-7 cells, containing a PI3KCA mutation, were among the most sensitive, with an IC_50_ of 2.25 µM. In contrast, whilst MK-2206 (the most advanced clinical allosteric AKT inhibitor) demonstrated similar levels of potency in MCF-7 cells, cytotoxicity was observed in 4 of the 6 normal cell lines tested (EC_50_ values less than 20 µM; (Fig. 3A)). The anti-proliferative effect of ALM301 was further confirmed in the MCF-7 cell line using live cell imaging: 5 µM of ALM301 completely abrogated cell proliferation over a 7d time course (Fig. 3B).

### ALM301 inhibits AKT phosphorylation and modulates downstream signalling in vitro

To further characterize the effects of ALM301 on cellular signalling, the phosphorylation levels of AKT (Ser473) were measured by ELISA in a sensitive cell line identified in previous experiments (MCF-7). Western blot analysis of MCF-7 cells treated with ALM301 further confirmed the time and concentration dependent inhibition of both pAKT and pGSK3β in vitro (See Fig. 3C for summary, and Supplementary Fig. S5A–G for raw data). Inhibition of AKT phosphorylation was observed 1 h after treatment of cells with 1 µM of ALM301 and was sustained up to 48 h. ALM301 was determined to inhibit phosphorylation of AKT with an EC_50_ of 0.47 µM compared to an EC_50_ of 2.55 µM for MK-2206 (Supplementary Fig. S3A–C).

Characterisation of the cytotoxic effect of ALM301 inhibition, using a clonogenic assay, was performed in MCF-7 cells over a concentration range of 0.001–10 µM, (IC_50_ = 0.1 µM for inhibition of colony formation, Fig. 3D). Furthermore, cell-cycle analysis in the same cell line following treatment with ALM301 demonstrated an increase of sub-G0 population in a concentration dependent manner, indicative of cell death (Fig. 3E).

A synergy screen, using ALM301 and combination partners in a matrix design, was conducted to identify compounds which are synergistic in combination with ALM301. Synergy was calculated using the median concentration effect method using Calcusyn software as previously described^26^. This screen indicated that tamoxifen showed strong synergy in combination with ALM301 in MCF-7 cells (*p* = 0.01) (Fig. 3F and Supplementary Table S5). In line with this, combination of ALM301 with tamoxifen showed a 4.5 × increase in sub-G0 population when compared to ALM301 alone, indicative of cell-cycle arrest (*p* = 0.004) (Fig. 3G).

### Determination of PK/PD relationship in A549 tumours

**Figure 4:**
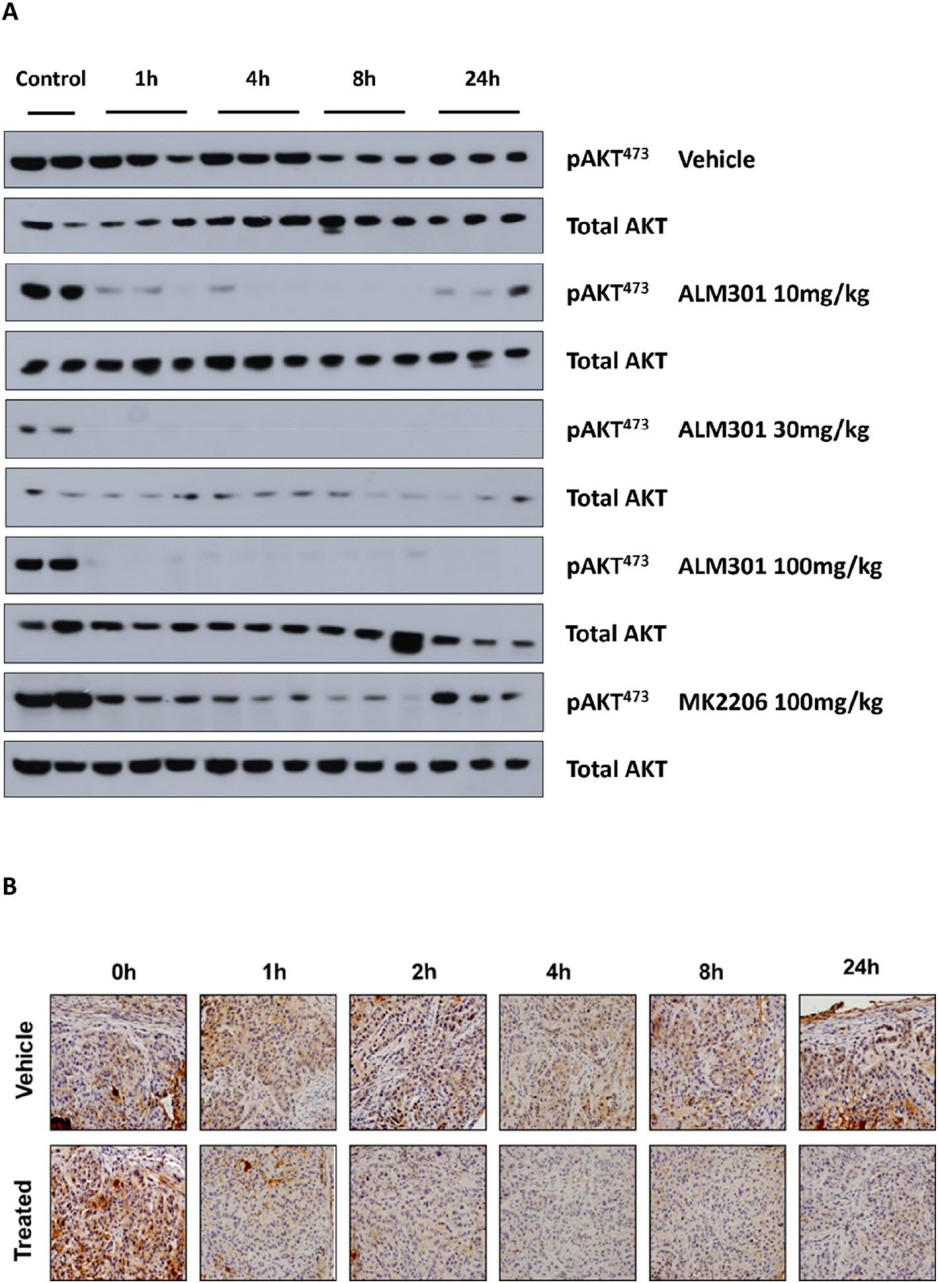

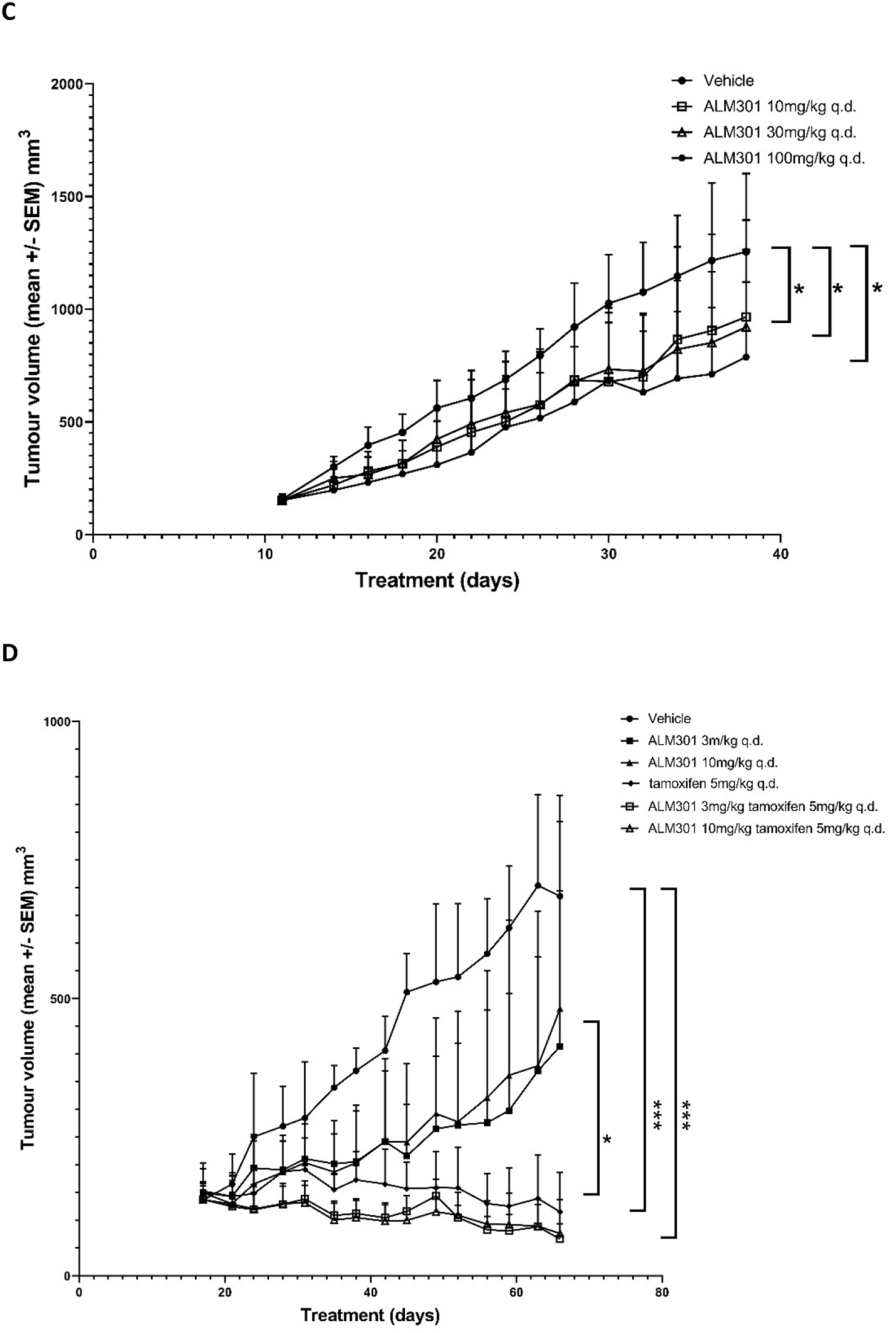
PK/PD and efficacy studies using development candidate ALM301 in A549 and MCF-7 xenograft models. (**A**) PK/PD relationship for ALM301 in A549 lung cancer xenograft model analysing levels of pAKT^S473^ in tumours using western blotting over 24 h after a single oral dose of either ALM301 (10, 30 or 100 mg/kg) or MK-2206 (100 mg/kg). (**B**) Representative immunohistochemistry sections of phospho AKT stained tumour samples (brown) treated with a single dose of either vehicle only or 30 mg/kg of ALM301 over 24 h period (**C**) Growth inhibition curves in an A-549 tumour xenograft model treated with either vehicle (p.o., q.d. × 28, n = 10/group) or ALM301 (p.o. 10/30 mg/kg q.d. × 28 or p.o. 100 mg/kg q.d × 3 then q.o.d. × 25, n = 10/group). Paired *t*-test was used to derive *p* values (n = 10/group), *p < 0.05, **p < 0.01, ***p < 0.001. (**D**) Growth inhibition curves of MCF-7 tumours treated with either vehicle (p.o., q.d. × 49, n = 10/group), ALM301 (p.o. 3 or 10 mg/kg q.d. × 49 n = 10/group), tamoxifen (p.o. 5 mg/kg q.d. × 49, n = 10/group) or ALM301 (p.o. 3 or 10 mg/kg q.d. × 49 n = 10/group) in combination with tamoxifen (p.o. 5 mg/kg q.d. × 49). Paired *t*-test was used to derive *p* values (n = 10/group), *p < 0.05, **p < 0.01, ***p < 0.001. ADME: Absorption, distribution, metabolism, excretion; DMPK: drug metabolism and pharmacokinetics; PK/PD: pharmacokinetics and pharmacodynamics.

In order to understand the relationship between ALM301 plasma exposure and in vivo AKT target engagement over time, a PK/PD study was carried out in a A549 lung cancer xenograft model. Briefly, plasma and tumour samples were collected at multiple timepoints over 24 h after administration of a single oral dose of ALM301 (10, 30 or 100 mg/kg) or MK-2206 (100 mg/kg) in BALB/c mice bearing A549 xenografts. ALM301 plasma concentrations and levels of pAKT^S473^ in tumours were measured using western blotting and immunohistochemistry (Fig. 4A, B and Supplementary Fig. S4A–C). ALM301 demonstrated dose-dependent increases in total plasma concentrations that resulted in almost total abrogation of measurable pAKT^S473^ in tumours at all timepoints over 24 h (Fig. 4A). In contrast, MK-2206 when dosed at 100 mg/kg orally in the same xenograft model showed incomplete inhibition of pAKT^S473^ over a 24-h time period (Fig. 4A). Tumour sections were collected following one dose of either vehicle or 30 mg/kg of ALM301 at noted time intervals (1 h, 2 h, 4 h, 8 h, 24 h). Tissue sections were subsequently stained with pAKT antibody (brown) staining and scored for pAKT staining using a scale of 1–4 (low to high) (Fig. 4B).

Subsequently, an anti-tumour efficacy study was carried out in an A549 lung cancer xenograft model using ALM301 orally dosed at 10, 30 and 100 mg/kg q.d*.* or q.o.d. (Fig. 4C). Modest tumour growth inhibition (TGI) of 23, 31 and 41% was observed for the three dosing groups of 10, 30 and 100 mg/kg respectively over the course of the study, in line with the relatively modest sensitivity of this cell line in vitro (IC_50_ = 43 µM). ALM301 was well tolerated in the 10 and 30 mg/kg dose groups with no body weight loss or adverse effects observed. Moderate bodyweight loss was observed in the 100 mg/kg dosing group that subsequently recovered after an amended dosing regimen of every other day was implemented (Supplementary Fig. S4D).

### ALM301 demonstrates efficacy as both single agent and in combination in a sensitive MCF7 breast cancer xenograft model

We next evaluated the inhibition of tumour growth following treatment with ALM301 in a more sensitive xenograft model. Given the high in vitro sensitivity of PIK3CA mutant MCF-7 cells to ALM301 both as single agent and in combination with tamoxifen (Fig. 3F,G), an MCF-7 xenograft model was selected. Once-a-day oral administration of ALM301 at either 3 or 10 mg/kg showed minimal tumour growth inhibition whereas combination treatment of ALM301 and tamoxifen demonstrated significant synergistic anti-tumour efficacy when compared to tamoxifen alone (*p* = 0.05) (Fig. 4D). Oral administration of tamoxifen (5 mg/kg q.d.) in combination with ALM301 (3 or 10 mg/kg q.d.) versus tamoxifen alone showed significant tumour regressions of 57% and 50% for the 3 and 10 mg/kg ALM301 combination dosing groups respectively (*p* = 0.0001 versus vehicle only group). In contrast, tamoxifen alone demonstrated tumour regression of only 24% during the course of the study. Importantly, ALM301 was well tolerated, both as monotherapy and in combination with tamoxifen, with no significant effects on animal body weight observed at any dose levels (Supplementary Fig. S4E).

## Discussion

The serine/threonine kinase AKT has been identified as a promising therapeutic target due to its critical role in multiple signalling cascades, including control of cell growth and proliferation, both of which are frequently dysregulated in cancer. In this study we highlight the development of ALM301, a novel, highly potent subtype-selective small molecule inhibitor of AKT1/2 and demonstrate its utility in preclinical xenograft models. ALM301 is highly selective across the kinome, including against members of the AGC family of kinases, a feature often lacking in ATP competitive AKT inhibitors^27,28^. Notably, ALM301 did not demonstrate any inhibition versus the additional 21 PH domain containing kinases assayed, differentiating ALM301 from other AKT inhibitors including ATP competitive inhibitors that have reported off-target liabilities^26^. ALM301 did not inhibit any of the PH domain null isoforms, in keeping with previously published data on allosteric inhibitors^29,30^. The potency of ALM301 was confirmed using in vitro kinase assays in which both full length AKT1 and AKT2 were potently inhibited, with potency versus AKT3 reduced by over 20-fold. ALM301 potently inhibited both AKT and GSK3β phosphorylation in vitro^31^. Given the differential expression of AKT isoforms 1 and 2 and the low levels of expression of AKT3, the development of AKT 1/2 isoform selective inhibitors may be advantageous in cancer settings where the relative abundance of each isoform is specific to each cancer type. In this context, increased activation of AKT1 has been reported in both breast and colorectal cancer, whilst pancreatic cancers frequently have increased expression of AKT2. Importantly, inhibition of specific AKT isoforms has been shown to have differential effects on tumour vascularisation, invasion and metastasis: inhibition of AKT3 in triple negative breast cancer was shown to increase migration in vitro, and subsequent studies demonstrated combined inhibition of either AKT1/3 or AKT2/3 resulted in increased metastasis formation in vivo^32^. Additional studies in the colorectal cancer cell line DLD-1 have demonstrated that combined knockout of AKT1 and 2 provided the most significant reduction in migration rate compared to either alone^33^.

It has been reported previously that effective treatment with PI3K/AKT/mTOR inhibitors is more favourably associated with PIK3CA mutation^34^. In agreement with these findings, the most significant anti-proliferative effects of ALM301 were observed in MCF-7 cells in vitro, a cell line containing a PIK3CA mutation. MCF-7 cells are estrogen receptor positive, a trait shared with over 70% of human breast cancers^35^. Despite treatment with surgery and anti-estrogen therapy, a significant proportion of patients will relapse with aggressive disease which is resistant to tamoxifen. Previous studies demonstrated the correlation between AKT phosphorylation in tamoxifen resistant cell lines^36^. Additionally, fulvestrant hyper-activation of PIK3CA mutation confers adaptation of estrogen receptor positive cells to estrogen deprivation, an effect which is abrogated with treatment of endocrine inhibitors^37^. We have demonstrated the synergistic effects of ALM301 in combination with tamoxifen and showed that combination therapy resulted in improved efficacy both in vitro and in vivo compared to monotherapy alone, which may represent a valuable therapeutic avenue for breast cancer patients currently resistant to long term estrogen deprivation therapy.

In conclusion, ALM301 is a differentiated, selective inhibitor of AKT isoforms 1 and 2 with a pharmacological profile fully consistent with its mechanism of action and ADME and PK properties suitable for further development. This compound demonstrated efficacy in tumour growth models and was well tolerated in vivo, demonstrating its potential as a clinical AKT inhibitor. We anticipate that this compound will allow further interrogation of the role of distinct AKT subtypes in human disease.

## Methods

### Compounds

ALM301 (6-(4-((1*S*,3*S*)-1-amino-3-hydroxycyclobutyl)phenyl)-1-ethyl-7-phenyl-1*H*-pyrido[2,3-*b*][1,4]oxazin-2(3*H*)-one (Fig. 1) and compounds **1** and **2** (Fig. 1) were all identified and synthesised at Almac Discovery Ltd as described previously (ALM301, compound **1** and compound **2** are Examples 139, 30 and 31 respectively in patent WO2011077098. MK-2206 (Supplementary Fig. S3A) was synthesised according to published patent procedures and tamoxifen was purchased from commercial providers.

### Kinase inhibition assessment

Biochemical analysis was performed using kinases obtained from Millipore (AKT1, ∆AKT1, ∆AKT2, ∆AKT3) and Signal Chem (AKT2 and AKT3). The specific activity of AKT 1,2 and 3 was determined to be 114 nmol/min/mg, 44 nmol/min/mg and 200 nmol/min/mg respectively as per activity assay protocol. ALM301 was profiled using the Fluorescent Polarisation (FP) iMAP Screening Express kit (Molecular Devices) using the FAM-Crosstide substrate [5FAM-GRPRTSSFAEG-OH] (Molecular Devices) against the three isoforms of AKT and their PH domain null mutants. Compounds were dissolved in DMSO prior to screening at 14 point titration series in the range (50 µM–50 pM). Fluorescence polarisation was measured using a Biotek Synergy 4 Hybrid microplate reader. Percentage inhibition of kinase activity was calculated and IC_50_ values derived using non-linear regression on GraphPad Prism. Competition of ALM301 with ATP was determined using AKT1 under increasing ATP concentrations up to 1000 µM.

### Kinase selectivity profiling

Off-target kinase activity was performed using the SelectScreen kinase profiling service against either mini 50 kinase or full 450 kinase panels using the Z’lyte and Adapta kinase activity platforms (performed by Thermofisher).

### In vitro proliferation assay

MCF-7, T47D, NCI-H460, HCT116, LNCaP, A431, PC3, PANC-1, A549, HT29M, SKOV-3, Du145, HMEC-1, Hs888Lu, Hela, MDA-MB-231 and SW480 cells were obtained from American Type Culture Collection (ATCC). A2780, PNT2, U251MG, HFFF2 and Fr2 cells were obtained from Public Health England European Collection of Authenticated Cell Cultures (Phe ECACC). InMyoFib and NHLF cells were acquired from Lonza. Cells were then incubated with increasing concentration of inhibitor (1 nM to 100 µM) for 72 h at 37 °C. Cell viability was determined using the CellTiter-Glo Luminescent Cell Viability Assay (Promega). IC_50_ values were derived as previously stated.

### In vitro incucyte assay

MCF-7 cells were obtained from American Type Culture Collection (ATCC). Cells were then incubated with increasing concentrations of inhibitor (1 nM to 100 µM) for 72 h at 37 °C. Cell confluency was measured over time using an Incucyte Zoom S3 using the basic analyser processing analysing tool. Percentage confluence values were calculated and IC_50_ values were derived as previously stated.

### Enzyme-linked immunosorbent assay (ELISA)

Cells were treated with AKT inhibitors, 1 nM to 10 µM as indicated in the figures, for 24 h at 37 °C. Cells were then washed with PBS and lysed. The subsequent supernatant was assayed using a GSK3β (pS9) (Invitrogen, Life Technologies) ELISA. Phospho-AKT levels were detected using the phospho-AKT (S473) immunoassay (R&D Systems) as per manufacturer’s protocol. Percentage inhibition of phosphorylation status was calculated by comparing phospho-AKT levels to total AKT levels with each treatment and IC_50_ values derived using non-linear regression on GraphPad Prism.

### Western blot analysis

AKT inhibitor treated cell lysates were obtained using RIPA lysis buffer supplemented with protease and phosphatase inhibitor tablets (Roche). Protein samples (30 µg/lane) were run by SDS-PAGE and transferred to Hybond C extra nitrocellulose membranes (Amersham Biosciences) before incubation with the following primary antibodies; pAKT, total AKT, pGSK3β, total GSK3 β, p(Cell Signaling Technologies), Actin (Sigma) or Vinculin (Santa Cruz). Primary antibodies were diluted at 1:1000 (except Actin which was diluted 1:5000). Secondary antibodies were diluted 1:10,000. Blots were visualised using Amersham ECL plus western blot detection system (GE Healthcare) or Odyssey CLx system (LI-COR).

### Flow cytometry-cell cycle

MCF-7 breast cancer cells were treated with increasing concentrations of ALM301 (0.1–100 µM) for 72 h. Post treatment, media containing detached cells was collected for each sample and the remaining adherent cells detached using 0.1% EDTA in PBS. Resulting cell suspensions were incorporated with associated media, centrifuged to form a pellet, washed with 1% FCS in PBS and fixed using ice-cold ethanol. Samples were stored at 4 °C for 24 h before staining with propidium iodide (10 µg/mL) (Sigma) containing 2.5 mg/mL RNase A (Qiagen) for 30 min at 37 °C and stored overnight at 4 °C before analysis. A minimum of 10,000 cells per sample were analysed for DNA content using a FACSCalibur flow cytometer and CellQuest Pro software (Becton Dickinson).

### Kinetic solubility assay

Compound solubility was tested in Dulbecco A PBS buffer at pH7.4 in Millipore MultiScreen Solubility Filter plates. Analysis was carried out on a Bioteck Synergy 4 plate reader (240–400 nm) and solubility quantified against a 5-point calibration curve.

### LogD_7.4_ assay

Compounds (100 µM) were shaken at 300 rpm at room temperature for 4 h in a biphasic system of octanol and PBS buffer pH 7.4 in a 96-well plate in triplicate. The relative drug concentration in each phase was then determined by the peak area measurement from LC-UV analysis on an Agilent 1260 chromatography instrument (UV detection at 254 nm and 220 nm). LogD is calculated as follows: LogD = Log[peak area of compound in octanol x injection volume of buffer phase/peak area of compound in buffer x injection volume of octanol phase].

### In vitro microsomal stability assay

Compounds (final concentration = 1 µM; final DMSO concentration = 0.1%) were incubated in 0.1 M phosphate buffer at pH 7.4 with liver microsomes (human, mouse, rat or dog; 0.5 mg of protein/mL) at 37 °C. Reactions were started by addition of NADPH in 0.1 M phosphate buffer pH 7.4 (final concentration 1 mM). Reactions were quenched by addition of ice-cold methanol and frozen. The supernatants were analysed by LC/MS/MS on a Waters Acquity I-Class coupled to a Waters Xevo TQD mass spectrometer. A Waters BEH C18 2.1 × 50 mm 1.7 µm column was used and mobile phases consisted of water and methanol containing 0.1% formic acid as modifier. Analysis was by multiple reaction monitoring and conditions were optimised for each test compound.

### Ethics declaration for in vivo studies

The rat pharmacokinetic study was outsourced to the CRO Huntington Life Sciences. The A549 PK/PD and efficacy study was outsourced to the CRO CrownBio. The MCF7 efficacy study was outsourced to the CRO BioDuro. All experimental protocols for in vivo studies were approved by the respective institutional ethics committees of either Huntingdon Life Sciences, Crown Biosciences or BioDuro. All methods were carried out in accordance with relevant guidelines and regulations, and are reported in accordance with ARRIVE guidelines.

Healthy, experimentally-naïve laboratory animals were purchased by the CROs from approved vendors. Sprague–Dawley CD albino male rats, BALB/c nude male mice, and SCID female mice were inspected by veterinary teams upon arrival, acclimated for one week, and housed in a specific pathogen-free setting with 12-h light–dark cycles with controlled temperature (20–26 °C) and humidity (40–70%) ranges. Rats were housed in groups of 3 and mice in groups of 5. Whenever possible, animals were not housed singly. All animals were housed in solid floor cages with access to food and water ad libitum and checked 1–2 times daily. Animals were cared for by trained husbandry personnel and procedures were conducted by trained scientists. All in vivo studies were performed in accordance with local and national animal welfare authorities. Studies performed in the United Kingdom were performed to the standards of the United Kingdom Animals (Scientific Procedures) Act 1986 and Directive 2010/63/UE European Convention for the Protection of Vertebrate Animals used for Experimental and Other Scientific Purposes under Home Office licences and by trained, licensed staff. Studies performed in China were performed according to the guidelines approved by the Institutional Animal Care and Use Committee (IACUC) of either Crown Bioscience or BioDuro following the guidance of the Association for Assessment and Accreditation of Laboratory Animal Care (AAALAC). All experiments were reviewed by local institutional Ethical Review Boards and conducted in compliance with good veterinary and animal husbandry practices. All experimental protocols were reviewed and approved before experiments were started. Dosing (5 mL/kg for *p.o.* dosing (10 mL/kg for rat *p.o.*) and 1 mL/kg for *i.v.* dosing) and sampling volumes (minimised to ensure no more than 10% of blood volume was taken over a 28-day period) were the lowest used to achieve the scientific objectives. Doses were calculated based on the bodyweight of the animal on that day. In line with the Principles of the 3Rs, the number of animals used was the lowest to achieve the scientific purposes. Study technicians were not blinded to the test agent and corresponding experimental dosing groups. At the end of the experiment, animals were euthanised using either an overdose of anaesthetic (either pentoject or euthatal) or exposure to a slow rising concentration of CO_2_, with confirmation of death using an approved humane second method.

### Pharmacokinetic study in Sprague–Dawley rats

Eight rats were purchased and 6 used on the PK study (3 per administration route). The remaining rats were purchased in case of mis-dosing events and were used to obtain blank biological matrix. Compounds were administered as single *i.v.* doses at 1 mg free base/kg or single oral doses at 5 mg free base/kg, with blood samples obtained from three rats per dose route, at 9 time-points out to 24 h post-dose. Both the intravenous and oral doses were prepared at a concentration of 1 mg free base/mL in a mixture of 5% DMSO/95% saline. Blood samples were centrifuged to give plasma. Plasma proteins were precipitated, and compounds extracted by addition of six volumes of acetonitrile containing warfarin as internal standard. Samples were centrifuged at 4000 rpm for 30 min and the supernatants were analysed on a Waters Xevo TQ mass spectrometer. Multiple Reaction Monitoring (MRM) methods were developed for each test compound using Waters IntelliStart software.

Quantification of test compounds was by extrapolation from calibration lines prepared in control rat plasma and analysed concurrently with experimental samples.

Non-compartmental analysis was performed using the software package PK Solutions 2.0 from Summit Research Services to produce the PK parameters. AUC values were calculated by the trapezoidal method.

Oral bioavailability was estimated by comparison of the drug concentration/time AUCs for individual animals after oral dosing with the mean AUC from *i.v.* doses.

### PK/PD and xenograft studies

For pharmacokinetic/pharmacodynamic (PK/PD) studies in A549 lung cancer xenografts, 150 male BALB/c nude mice aged 6–8 weeks and weighing approximately 18–22 g were inoculated with 1 × 10^7^ A549 tumour cells (ATCC) in 0.1 mL of PBS with Matrigel (1:1) subcutaneously into the right flank. Once the mean tumour size reached 150–250 mm^3^, the mice were randomised into treatment groups (18 mice per group) using a randomised block design (first, experimental animals are divided into homogeneous blocks according to their initial tumour volume; secondly, within each block, randomisation of animals to treatment groups was conducted so that each animal had the same probability of being assigned to a given treatment, thereby reducing systematic bias). Group sizes were devised based on previous pilot studies using a minimal numbers approach of n = 3 tumours per time-point (6 time-points per dose level). Mice received a single oral dose of ALM301 (either 10, 30 or 100 mg/kg), MK-2206 (100 mg/kg) or vehicle control. Tumour size was measured twice weekly in two dimensions using a caliper, and the volume expressed in mm^3^ using the formula: V = 0.5 (a × b^2^), where a and b are the long and short diameters of the tumour, respectively.

For efficacy in A549 xenografts, 90 male BALB/c nude mice aged 6–8 weeks and weighing approximately 20–26 g were inoculated subcutaneously in the right flank with 1 × 10^7^ A549 tumour cells (ATCC) in 0.1 mL of PBS with Matrigel (1:1) to aid tumour development. When the mean tumour size reached approximately 150 mm^3^, 10 mice were randomised to each experimental group, using a randomised block approach. Groups sizes were devised based on past experience of the provider. Treatment started on Day 11 after tumour inoculation with mice receiving ALM301 (either 10, 30 or 100 mg/kg) or vehicle control. Tumour size was measured twice weekly in two dimensions using a caliper, and the volume expressed in mm^3^ using the formula: V = 0.5 (a × b^2^), where a and b are the long and short diameters of the tumour, respectively.

For efficacy in MCF-7 xenografts, 150 SCID female mice aged 6–8 weeks and weighing approximately 18–22 g were implanted with 1 × 10^7^ MCF-7 tumour cells (ATCC) subcutaneously in the right flank. Each mouse had a 0.5 mg 17-β-estradiol 60-day release pellet implanted subcutaneously in the dorsal interscapular region the day before tumour inoculation. Once mean tumour size reached approximately 150 mm^3^, mice were randomised to either control or treatment groups (n = 10/group) using a randomised block approach. Groups sizes were devised based on past experience of the provider. Treatment started on Day 17 after tumour inoculation with animals in the monotherapy treatment groups receiving 3, 10, 30 or 100 mg/kg ALM301 or 5 mg/kg tamoxifen. Animals treated with combination therapy received either 3, 10 or 100 mg/kg of ALM301 plus 5 mg/kg tamoxifen. Tumour size was measured twice weekly in two dimensions using a caliper, and the volume expressed in mm^3^ using the formula: V = 0.5 (a x b^2^), where a and b are the long and short diameters of the tumour, respectively.

### X-ray crystallography

Human AKT-2 was expressed in insect cells and purified by affinity chromatography using a HIS-tag, followed by TEV-cleavage of the tag, negative affinity chromatography, anion exchange chromatography and size exclusion chromatography. The procedure yielded homogenous protein with purity > 95% as determined from Coomassie stained SDS-PAGE. The purified protein was used in crystallization trials including a standard screen with approximately 1200 different conditions as well as a pre-established condition (0.1 M MES, pH 6.5, 1.5 M NaH_2_PO_4_). Crystals of AKT-2 crystallized with compound 3 were flash-frozen and measured at a temperature of 100 K. X-ray diffraction data was collected at the Swiss Light Source using cryogenic conditions. The crystals belong to space group P 2_1_ 2_1_ 2_1_ and data was processed using XDS and XSCALE. Phase information was obtained by molecular replacement using a previously solved structure of AKT-2. Model building and refinement was performed according to standard protocols with CCP4 and Coot. Approximately 5.0% of measured reflections were excluded from the refinement procedure for the purposes of calculating Rfree. TLS refinement was performed using REFMAC5. Statistics for data collection and refinement of the final structure are shown in Supplementary Tables S3 and S4.

### Docking studies

The initial 3D conformation of ALM301, protonation state at pH 7.4 and partial charges were generated with Ligprep. ALM301 was docked into the AKT-2 crystal structure using Glide SP with standard settings. The top scoring pose was refined using MM-GBSA, with residues within 4.5 Å defined as flexible. The minimization sampling method and the constrain flexible residues option were used.

## Supplementary Information

Supplementary Information.

## Data Availability

The datasets used and/or analysed during the current study are available from the corresponding author on reasonable request. The authors confirm that the manuscript complies with the relevant digital image and integrity policies.
