# Supplementary material for: Identification and development of a subtype-selective allosteric AKT inhibitor suitable for clinical development
Source: Sci Rep. 2022 Sep 20;12:15715. doi: 10.1038/s41598-022-20208-5 (PMC9489722; doi:10.1038/s41598-022-20208-5)
Supplement: Supplementary file 1 — Supplementary Information. [file 41598_2022_20208_MOESM1_ESM.docx]

**Supplementary Materials**

**Supplementary Table S1**.

Comparison of ALM301 biochemical IC50 versus AKT1 using varying concentrations of ATP.

| **ATP Concentration (µM)** | **ALM301 IC_50_ (nM) vs. AKT1** |
| --- | --- |
| 150 | 121 |
| 250 | 125 |
| 500 | 157 |
| 1000 | 110 |

**Supplementary Table S2.**

Selectivity profiling of ALM301 versus a panel of 450 kinases. Kinases with greater than 70% inhibition at 10 µM fixed concentration shown only.

| **Kinase** | **% inhibition @ 10 µM** |
| --- | --- |
| AKT1 | 95 |
| AKT2 | 89 |
| p38 α | 84 |
| PDK1 | 77 |
| AKT3 | 72 |

**Supplementary Table S3**.

X-ray crystallography data collection and processing statistics.

| **X-ray source** | PXII/X10SA (SLS) |
| --- | --- |
| **Wavelength [Å]** | 1.0000 |
| **Detector** | PILATUS 6M |
| **Temperature [K]** | 100 |
| **Space group** | P 2_1_ 2_1_ 2­_1_ |
| **Cell: a; b; c; [Å]**  **α; β; γ; [°]** | 63.67; 113.88; 73.05  90.0; 90.0; 90.0 |
| **Resolution [Å]** | 2.32 (2.57-2.32) |
| **Unique reflections** | 23558 (6104) |
| **Multiplicity** | 4.1 (4.3) |
| **Completeness [%]** | 99.6 (99.5) |
| **R­_sym_­­ [%]** | 5.7 (44.1) |
| **R­_meas_­ [%]** | 6.5 (50.3) |
| **Mean(I)/sd** | 19.24 (3.76) |

**Supplementary Table S4**.

X-ray crystallography refinement statistics.

| **Resolution [Å]** | 61.49-2.32 |
| --- | --- |
| **Number of reflections (working / test)** | 22371 /1187 |
| **R­_cryst_ [%]** | 20.9 |
| **R­_free_ [%]** | 26.3 |
| **Total number of atoms:**  **Protein**  **Water**  **Ligand**  **Phosphate**  **MES**  **Glycerol** | 3158  147  33  15  12  6 |
| **Deviation from ideal geometry:**  **Bond lengths [Å]**  **Bond angles [°]**  **Bonded B’s [Å^2^]** | 0.011  1.55  3.3 |
| **Ramachandran plot:**  **Most favoured regions [%]**  **Additional allowed regions [%]**  **Generously allowed regions [%]**  **Disallowed regions [%]** | 91.9  6.9  0.3  0.9 |

**Supplementary Table S5**.

Cell proliferation combination indices of ALM301 in combination with Tamoxifen.

| **Drug** | Tamoxifen |  |
| --- | --- | --- |
| **Target** | ER |  |
| **ED_50_** | 0.81 | Synergy |
| **ED_75_** | 0.62 | Synergy |
| **ED_90_** | 0.55 | Strong Synergy |
| **r^2^** | 0.96 |  |

**Supplementary Figure Legends**

**Figure S1.**

Chemical structure and cyrstallographically determined binding mode of compound 3. (**A**) Chemical structure of compound 3. (**B**) Overall structure of compound 3 in complex with AKT2 binding approximately 10 Å away from the ATP binding hinge region (dark blue). (**C**) Close up of compound 3 bound in the AKT2 allosteric pocket showing amino acid interactions. Interaction of the PH domain with the catalytic domain was found to cause significant rearrangements of residues Arg184 to His209 and of the activation loop (Thr292 to Leu317), the latter of which included a shift to DFG-out. A combination of these movements creates an allosteric pocket in which compound 3 binds. The unsubstituted phenyl ring occupies the space vacated by Phe294 in the shift from DFG-in to DFG-out, approximately 10 Å away from the ATP binding hinge region (dark blue). The fused ring system is packed against the face of Trp80 on one side and the sidechains of Val271 and Leu266 on the other. The basic nitrogen attached to the cyclobutyl ring forms a salt bridge with Asp275, a hydrogen bond with the backbone carbonyl of Tyr273 and potentially also interacts with the π system of the Tyr273 sidechain. The hydroxyl group and sp^2^ ring nitrogen do not interact directly with the protein but rather with a network of water molecules within the site. (**D**) Compound 3 bound in allosteric pocket of AKT2 - amino acid interaction diagram. (**E**) 2F_o_-F_c_ map of compound 3 electron density contoured at 1σ.

**Figure S2.**

ALM301 is a highly selective AKT1/2 inhibitor versus a panel of kinases. (**A**) Kinome selectivity profile of ALM301 against a panel of over 450 kinases at a fixed concentration of 10 µM (Red lines = AKT1/2/3; orange line = P38α; green line = PDK1). (**B**) Follow-up biochemical titration of kinases demonstrating >70% inhibition at 10 µM in the 450 kinase panel against ALM301.

**Figure S3.**

Allosteric AKT clinical candidate MK-2206 profile. (**A**) Chemical structure of MK-2206. (**B**) MK-2206 biochemical potency against AKT1/2/3 isoforms. (**C**) Comparison of pAKT inhibition in the most sensitive cell line MCF-7 for ALM301 versus MK-2206 (24h treatment).

**Figure S4**

Inhibition of pAKT PD biomarker in A549 tumours and body weight effects of ALM301 dosing in A549 and MCF-7 xenograft models. PK/PD relationships at different time points (up to 24 hours) after single oral administration of ALM301 in BALB/c nude mice in A549 xenograft model at (**A**) 10 mg/kg, (**B**) 30mg/kg or (**C**) 100mg/kg. (**D**) Effect of oral dosing of ALM301 on bodyweight change in A-549 xenograft model. (**D**) Effect of ALM301 (alone or in combination with tamoxifen) on bodyweight change in MCF-7 xenograft model.

**Figure S5**

Raw data (full length, uncut gels) from Fig 3C. (**A**) Upper Band = pAKT, 1h and 4h timepoints; Lower Band = pGSK3β^Ser9^, 1h and 4h timepoints (**B**) Actin, 1h and 4h timepoints (**C**) Upper Band = Total AKT, 1h and 4h timepoints; Lower Band = Total GSK3β, 1h and 4h timepoints (**D**) Upper Band = Total AKT: 24h and 48h timepoints; Lower Band = pGSK3β^Ser9^, 24h and 48h timepoints (**E**) Actin: 24h and 48h timepoints (**F**) pAKT: 24h and 48h timepoints (**G**) Total GSK3β, 24h and 48h timepoints.

**Figure S6**

Raw data (full length, uncut gels) from Fig 4A. (**A**) ALM301 10mg/kg; pAKT: 1, 4, 8 and 24h timepoints; (**B**) ALM301 10mg/kg; Total AKT: 1, 4, 8 and 24h timepoints; (**C**) ALM301 30mg/kg; pAKT: 1, 4, 8 and 24h timepoints; (**D**) ALM301 30mg/kg; Total AKT: 1, 4, 8 and 24h timepoints; (**E**) ALM301 100 mg/kg; pAKT: 1, 4, 8 and 24h timepoints; (**F**) ALM301 100 mg/kg; Total AKT: 1, 4, 8 and 24h timepoints; (**G**) MK-2206 100 mg/kg; pAKT: 1, 4, 8 and 24h timepoints; (**H**) MK-2206 100 mg/kg; Total AKT: 1, 4, 8 and 24h timepoints.

**Supplementary Figure S1**

**A**

**
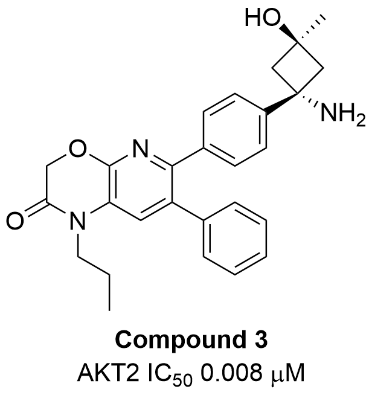
**

**B**


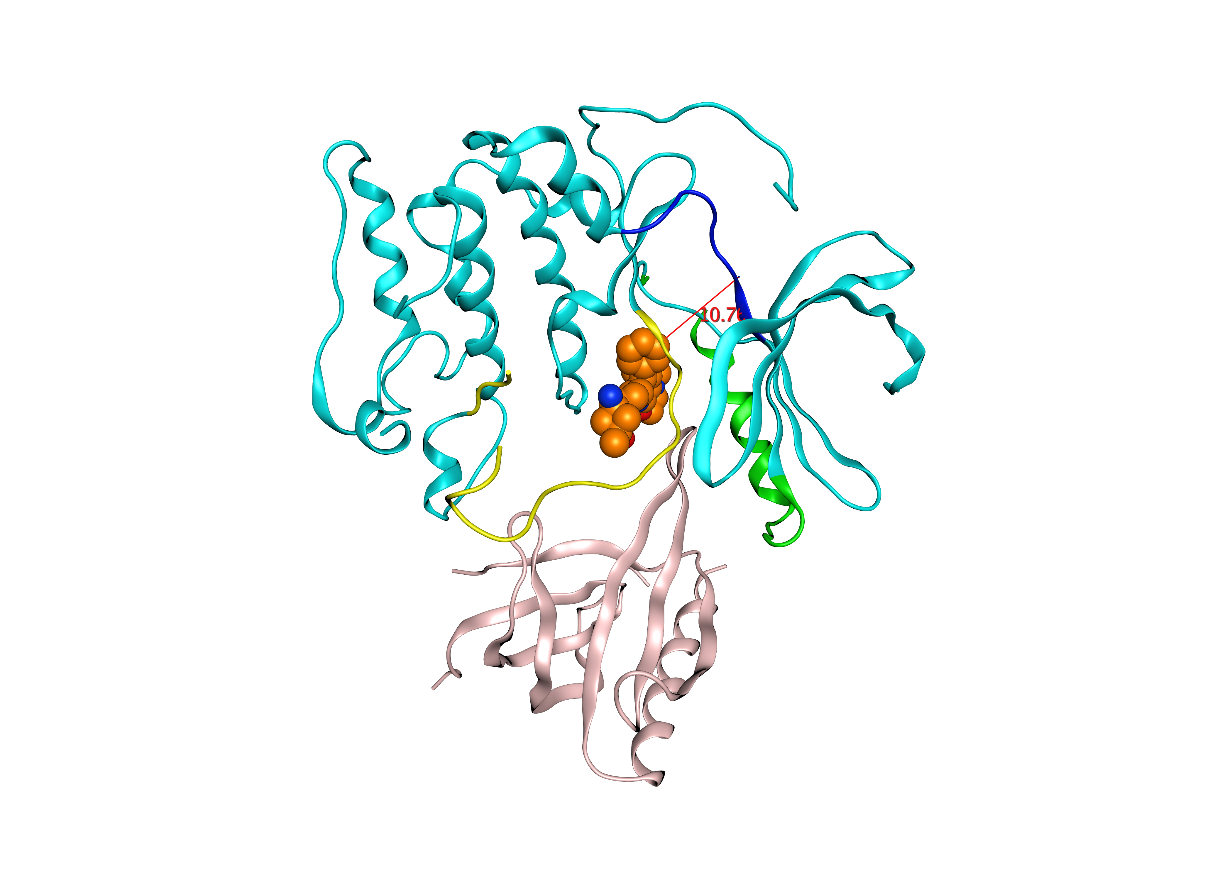


**C**


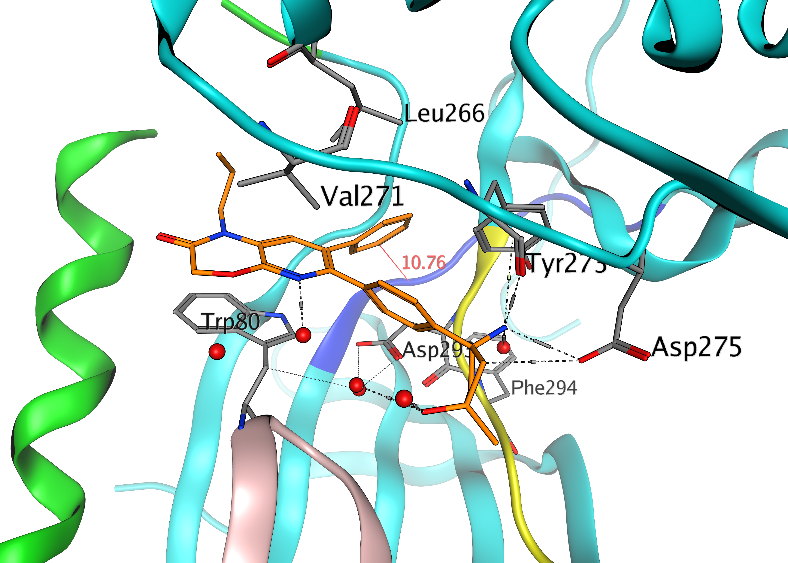


**D**


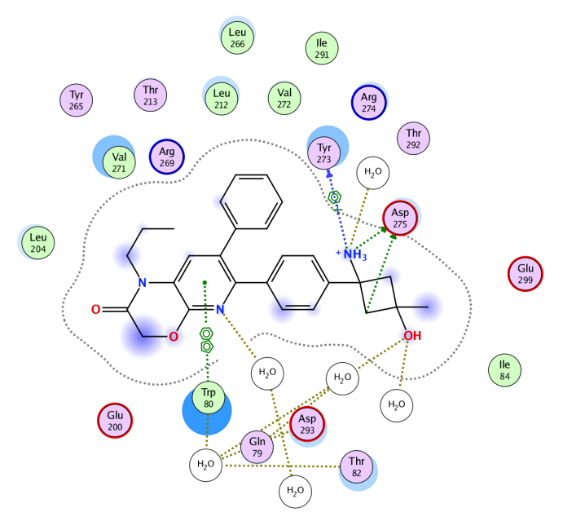


**E**


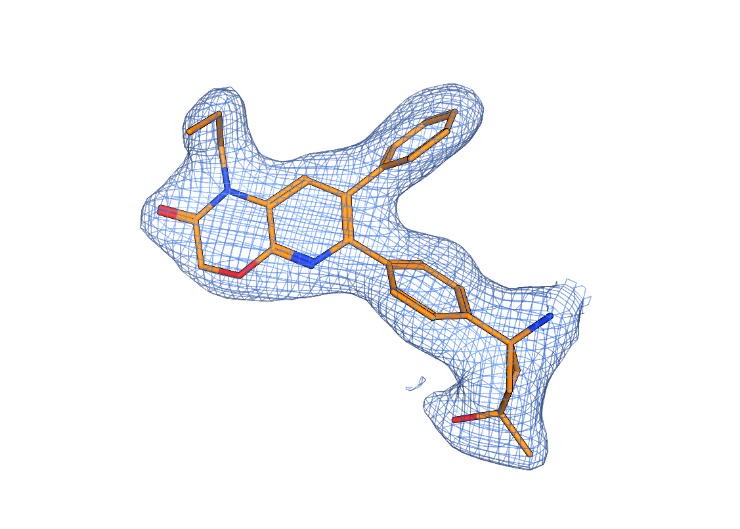


**Supplementary Figure S2**

**A**

**
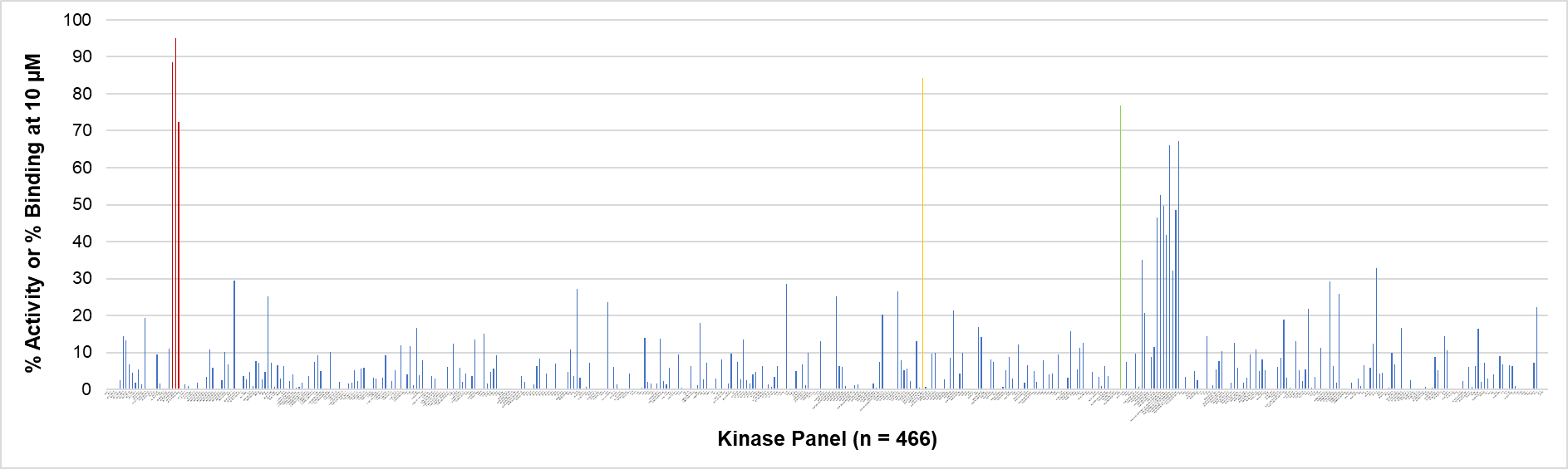
**

**B**


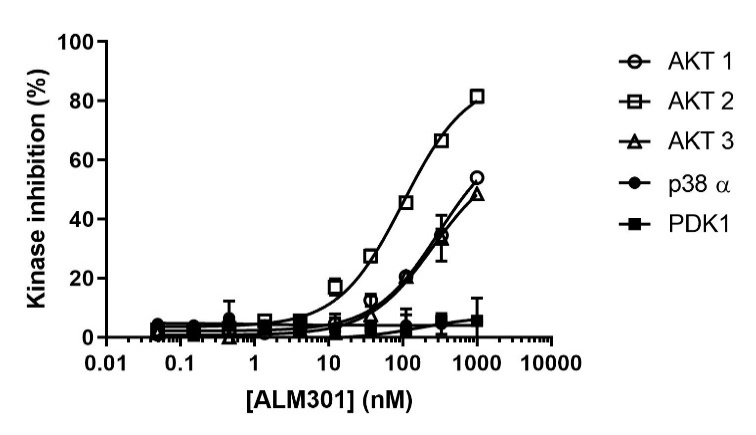


**Supplementary Figure S3**

**A**





**B**


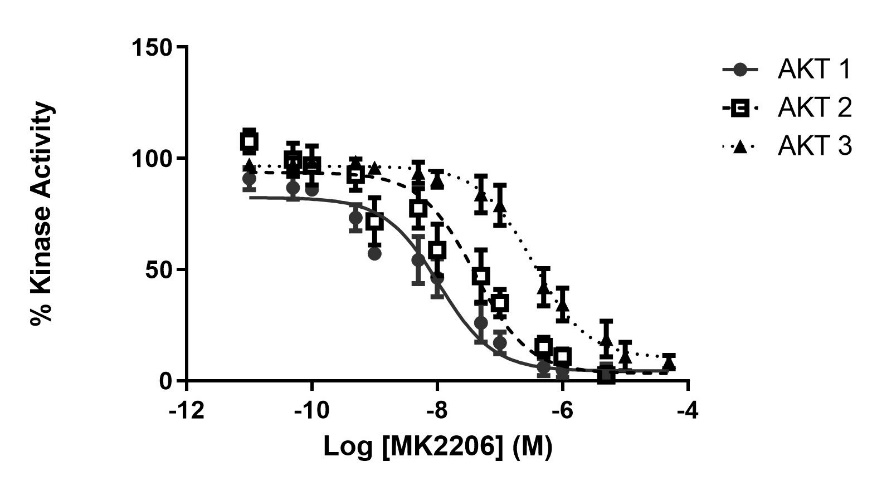


**C**


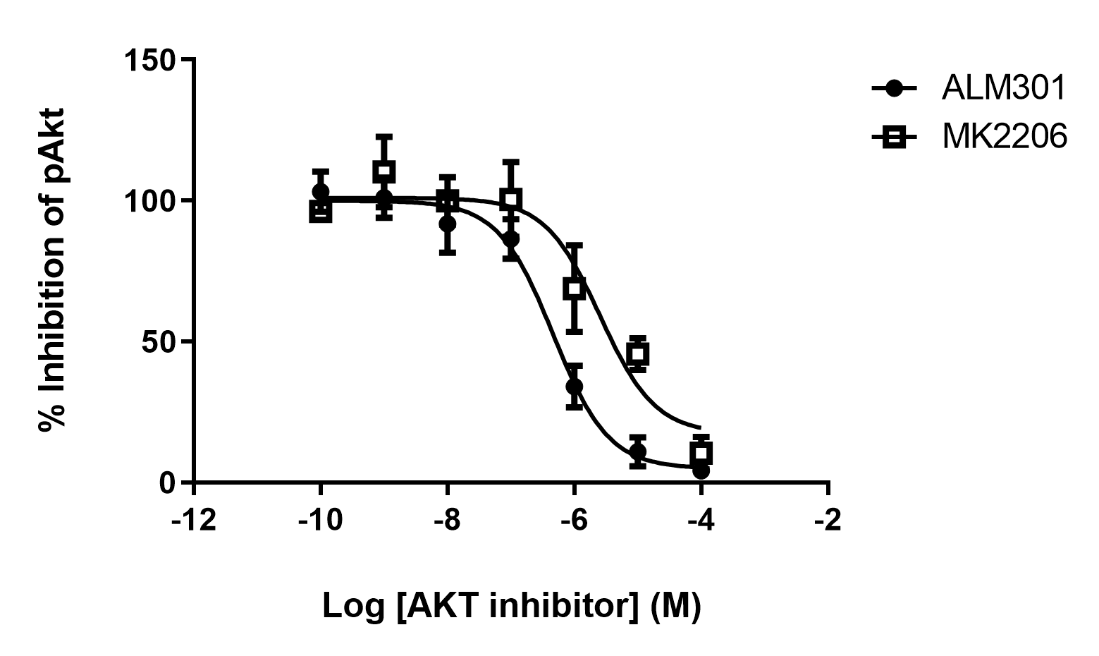


**Supplementary Figure S4.**

**A**


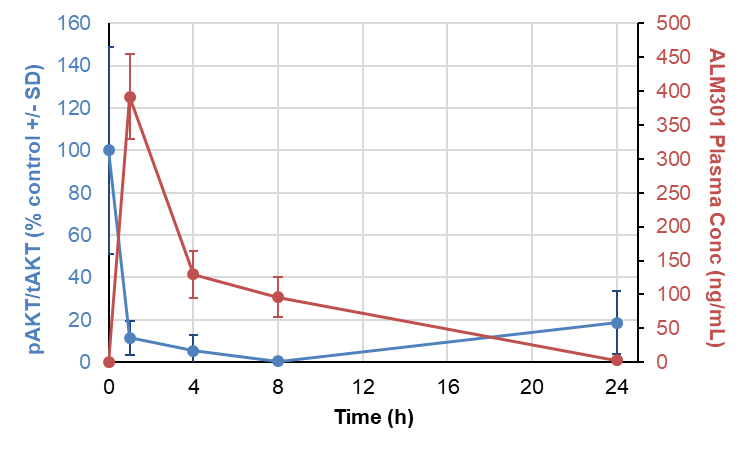


**B**


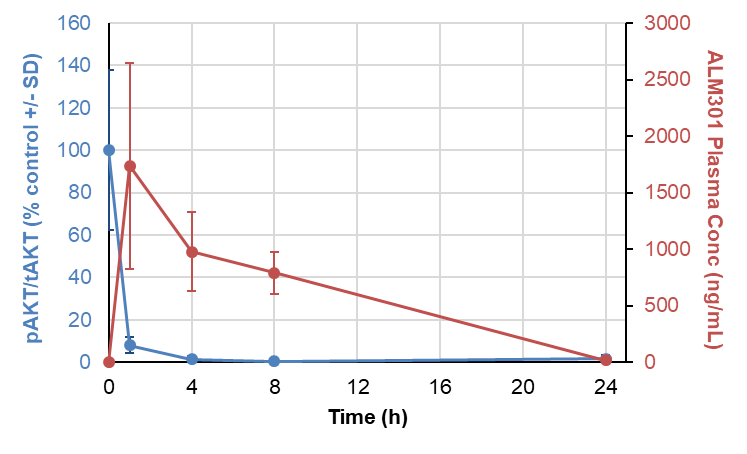


**C**


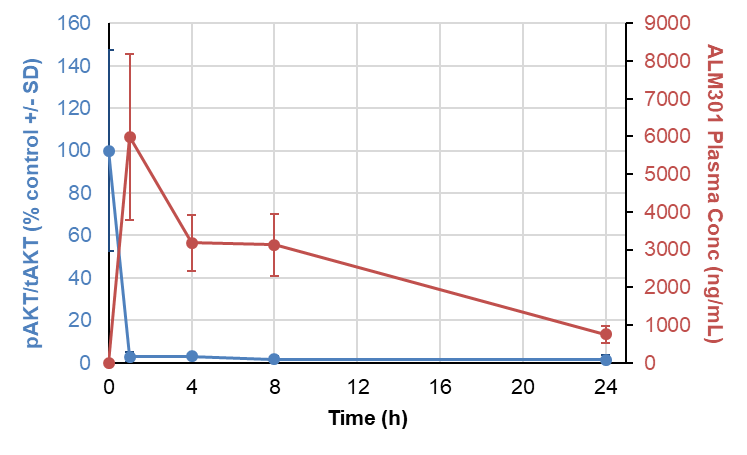


**D**

**
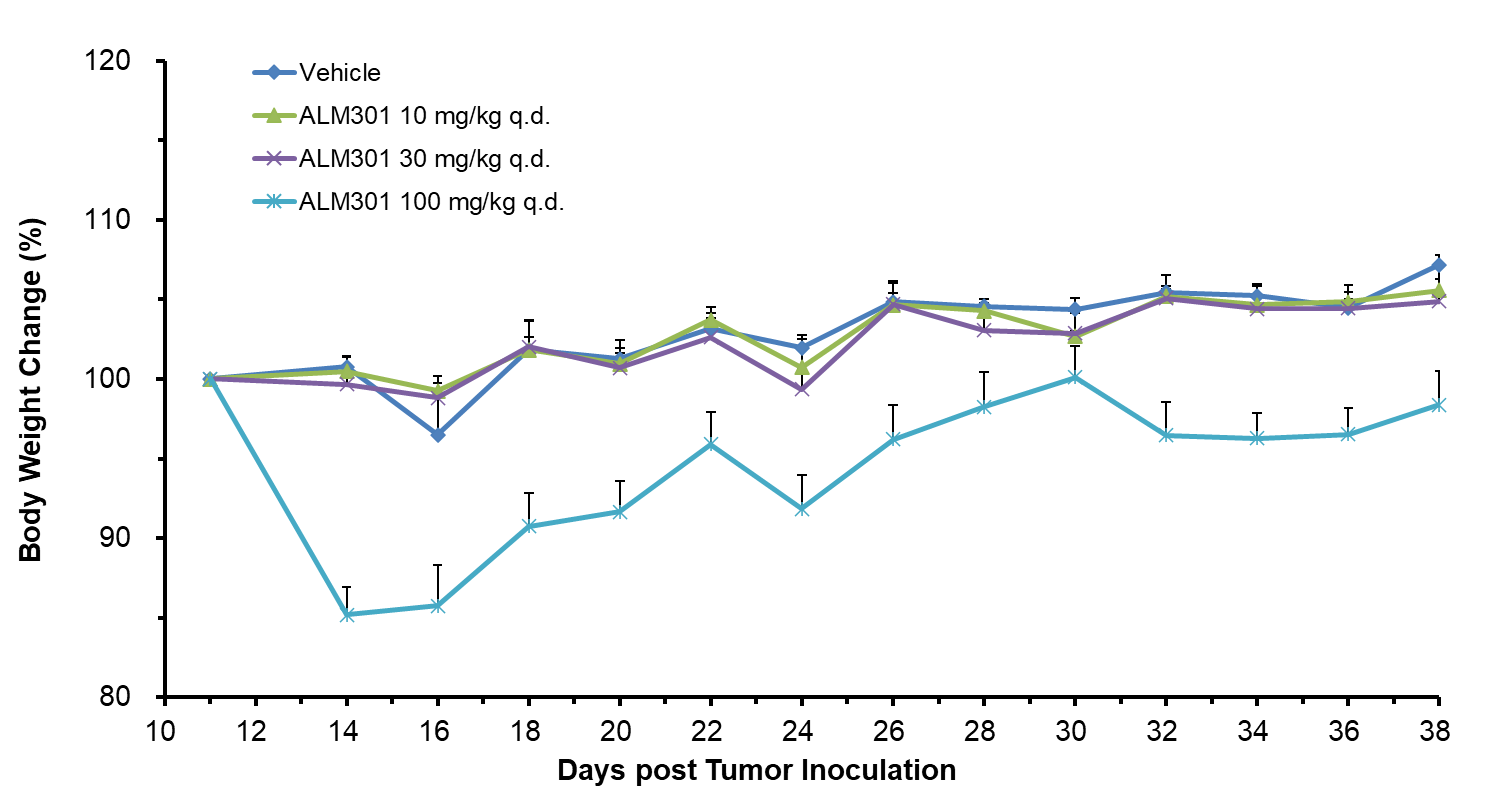
**

**E**


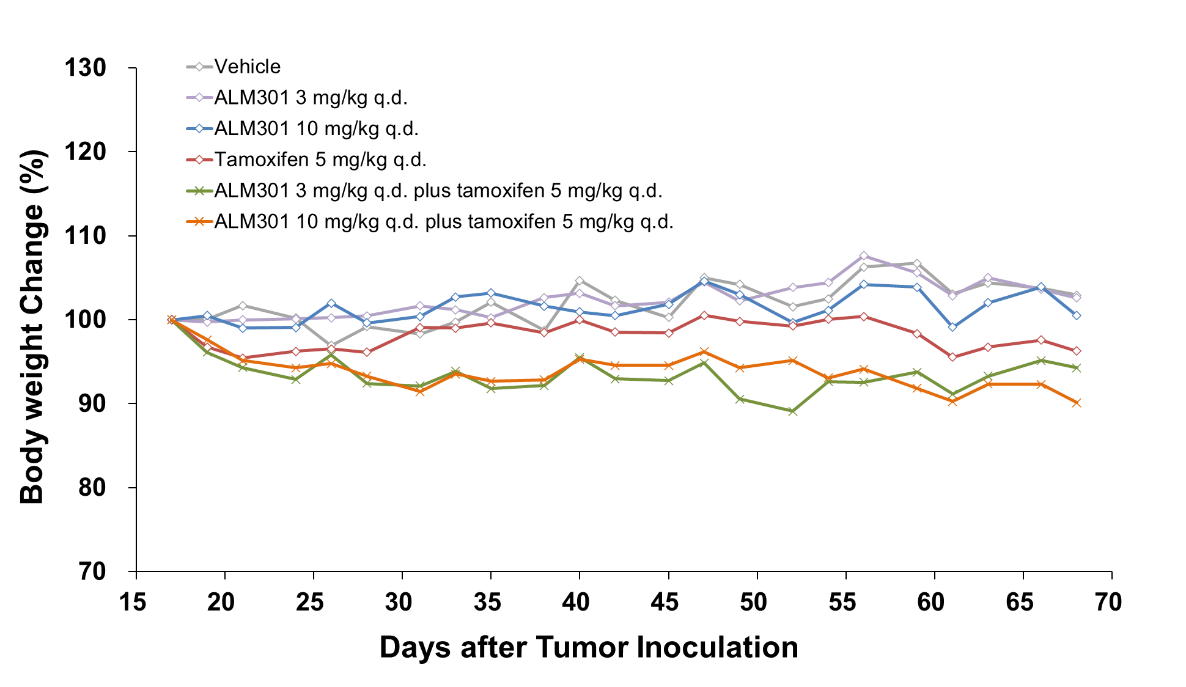


**Supplementary Figure S5**

**A**

 
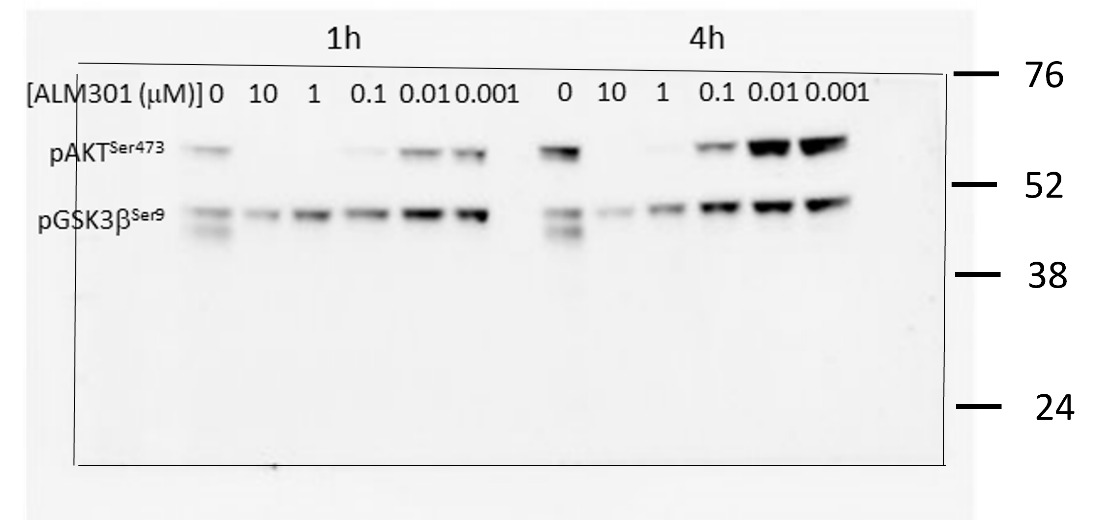


**B**


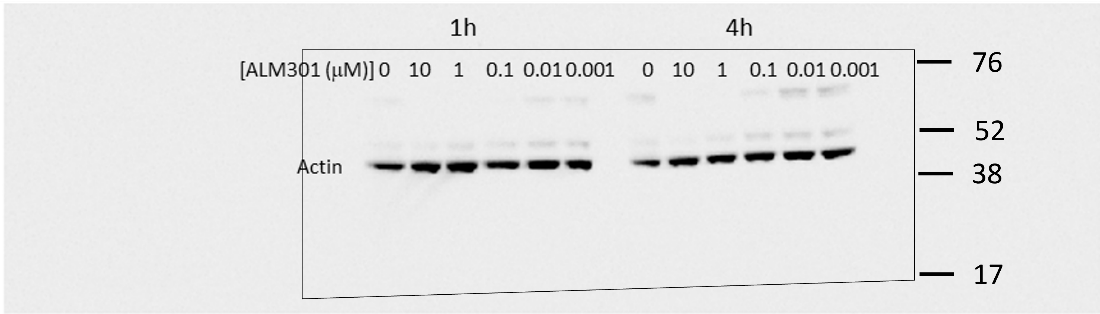


**C**


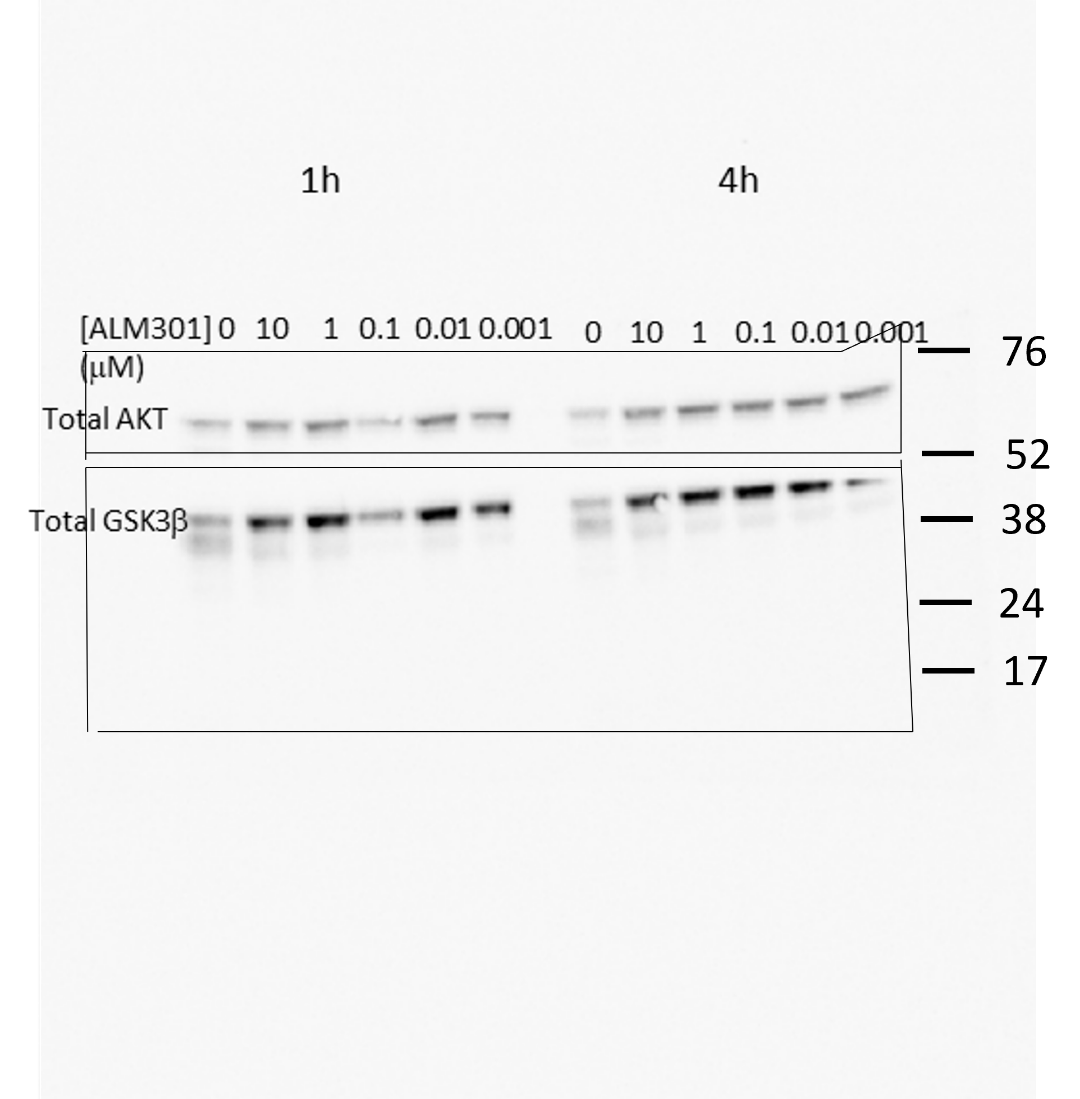


**D**


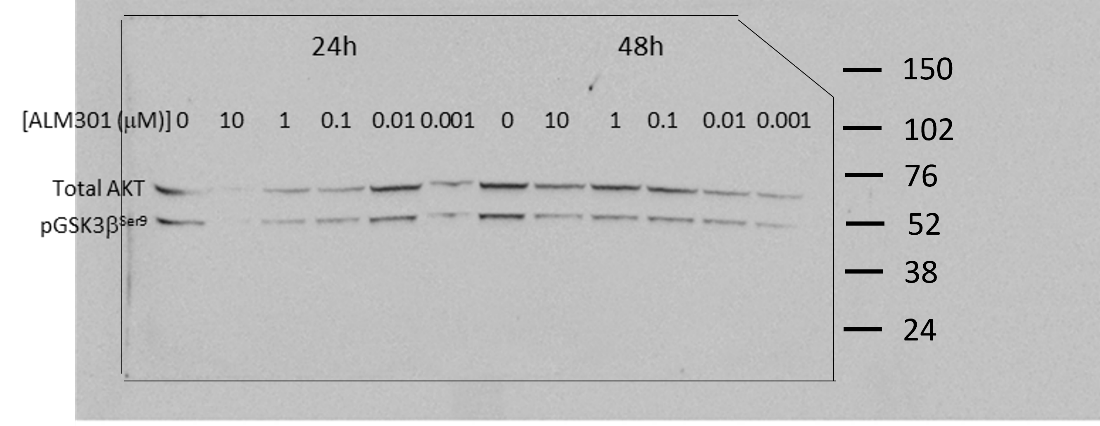


**E**


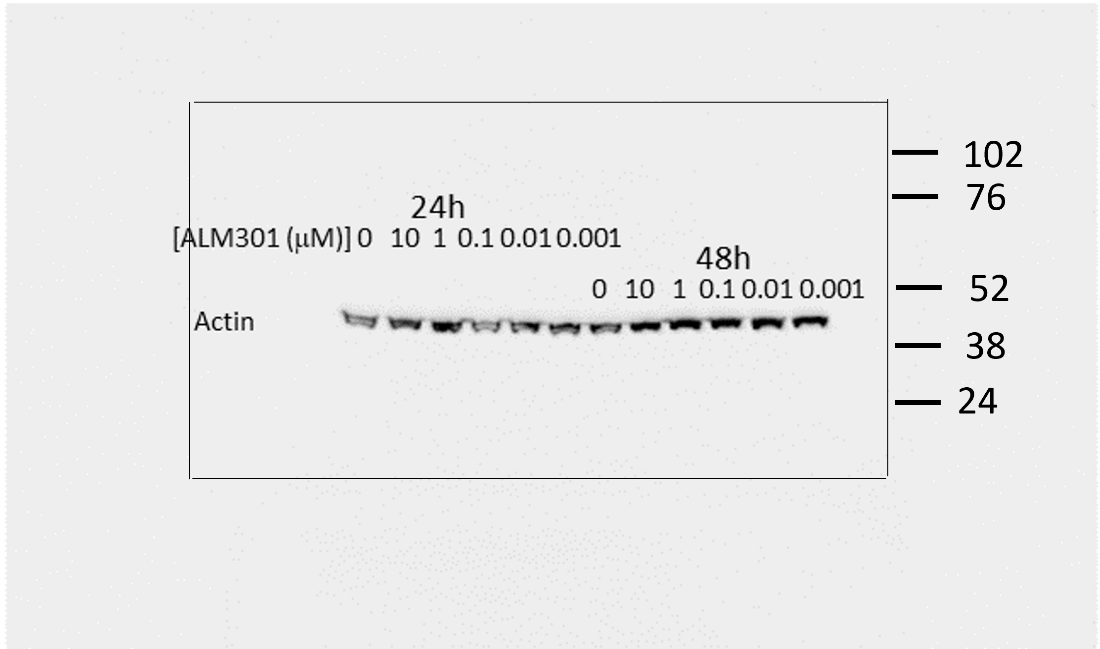


**F**


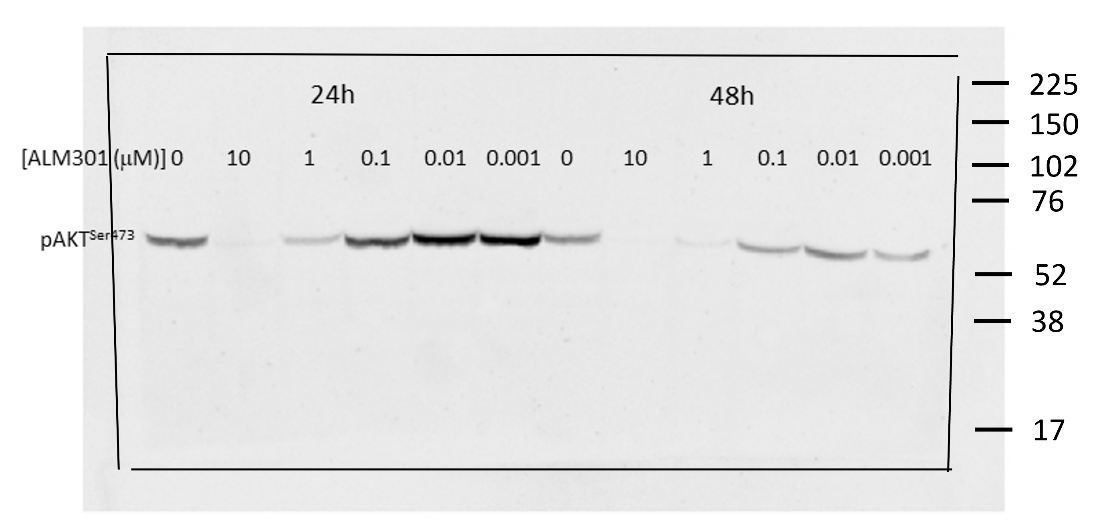


**G**


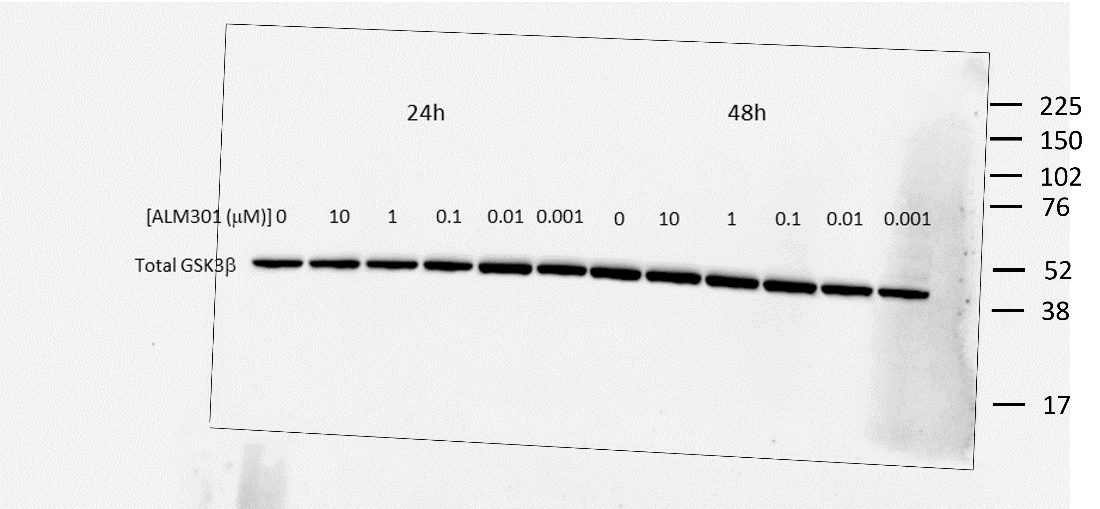


**Supplementary Figure S6**

**A**

**
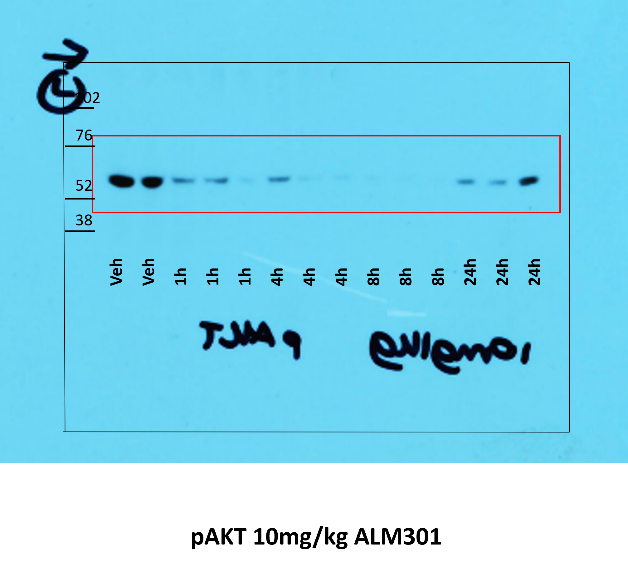
**

**B**

**
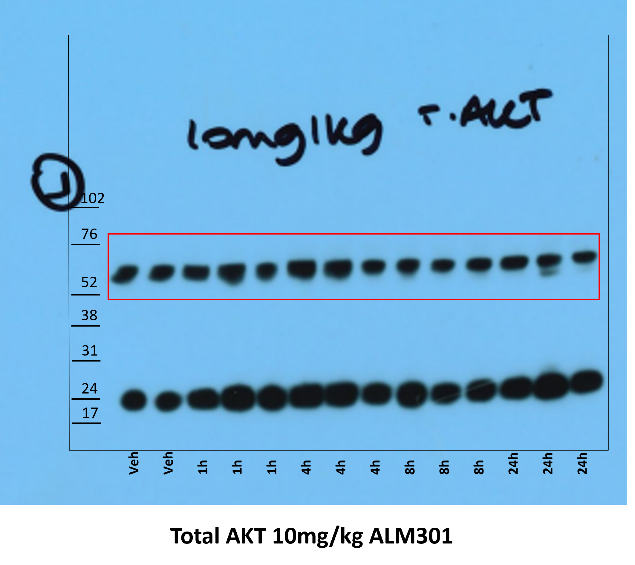
**

**C**

**
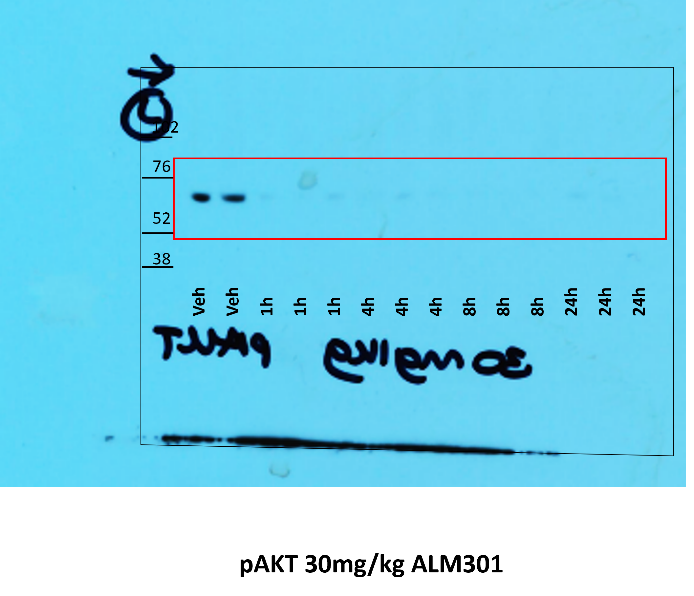
**

**D**

**
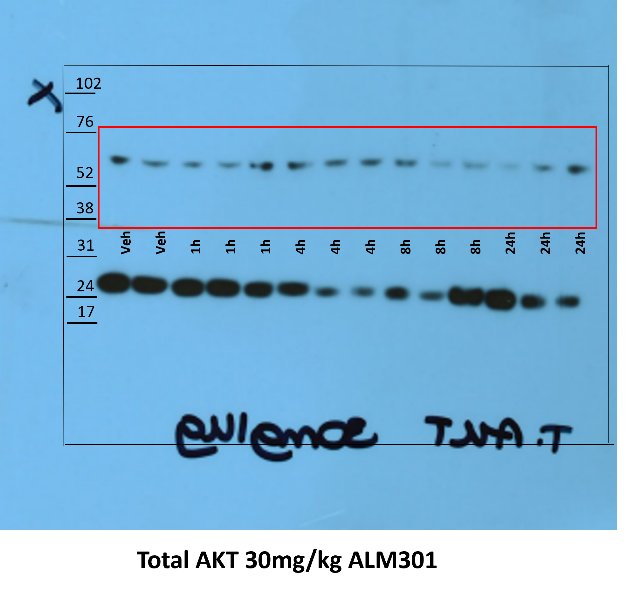
**

**E**

**
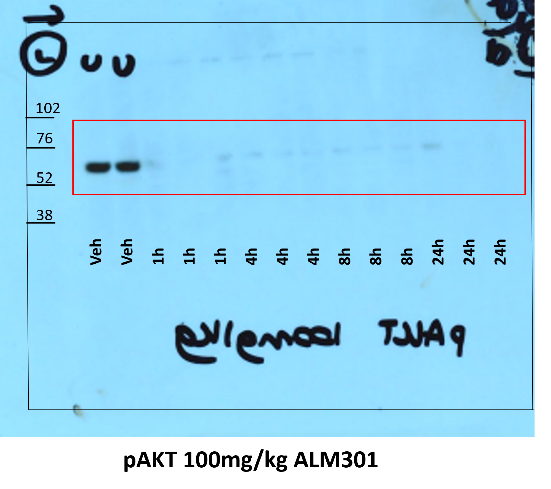
**

**F**


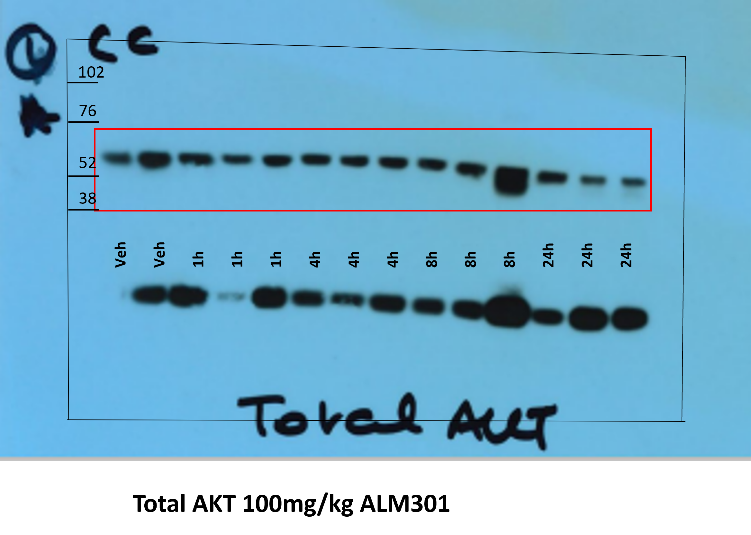


**G**


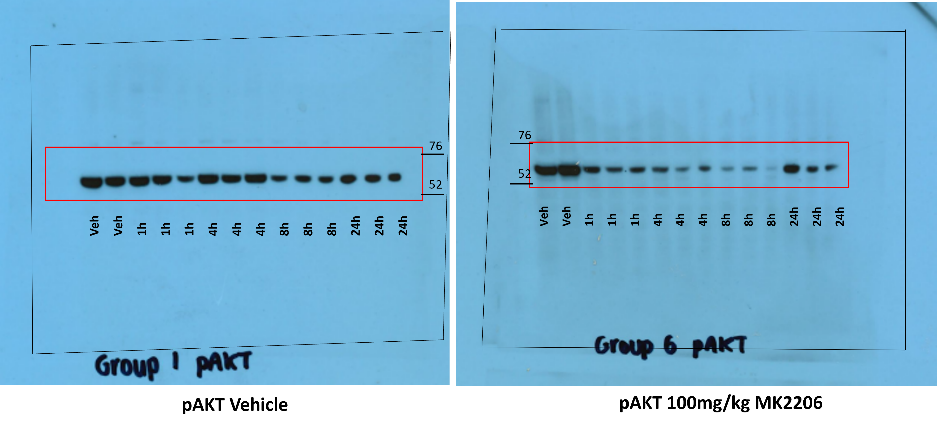


**H**

**
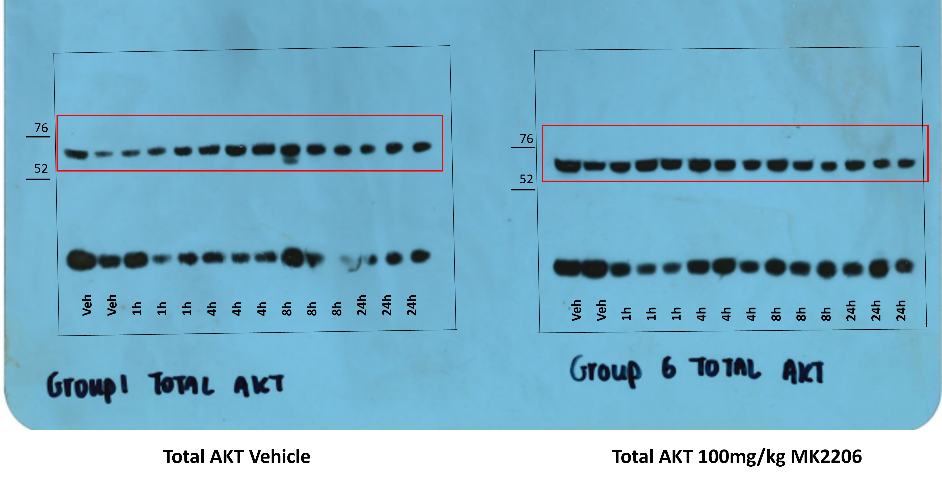
**
